# Reducing Barriers in Neurodiverse Schools—schAUT: A Program to Identify and Reduce Barriers for Autistic and All Students

**DOI:** 10.3390/bs16060949

**Published:** 2026-06-09

**Authors:** Lukas Hümpfer-Gerhards, Sabine Schwager, Mark Benecke, Stephanie Fuhrmann, Jana Kunert, Michel Knigge

**Affiliations:** 1 Faculty of Educational Sciences, Goethe University, 60323 Frankfurt am Main, Germany; 2 Faculty of Humanities and Social Sciences, Humboldt University, 10117 Berlin, Germany; sabine.schwager@hu-berlin.de; 3 White Unicorn e.V., 12623 Berlin, Germany; mark@benecke.com (M.B.); stephanie.fuhrmann@white-unicorn.org (S.F.); 4 Department Inclusive Education, Potsdam University, 14476 Potsdam, Germany; jana.kunert@uni-potsdam.de (J.K.); michel.knigge@uni-potsdam.de (M.K.)

**Keywords:** neurodiversity, School Organizational Development, barriers

## Abstract

This paper presents results from the project schAUT, a participatory research project initiated by Humboldt University Berlin, Goethe University Frankfurt a.M. and White Unicorn e.V., funded by the German Federal Ministry of Education and Research (BMBF; FKZ: 01NV2104). It aimed to identify and reduce barriers to learning and participation in mainstream schools, with a particular focus on autistic students. This paper introduces a questionnaire and a program to support School Organizational Development (SOD), aiming to provide equitable and accessible learning environments grounded in international frameworks on inclusive education. This study combines qualitative and quantitative approaches to examine the subjective experiences of barriers. We present data obtained through a multi-phase development and validation phase. The results show that neurodivergent participants generally experienced higher subjective barriers, although we observed that barriers affect neurotypical students as well, highlighting a subjective nature. We argue that these findings support neurodiversity as a relevant concept, especially in educational contexts. This supports Larrauri et al.’s Big-Tent approach to neurodiversity, emphasizing individual variability while acknowledging structural biases towards neurotypical norms in educational environments. The study highlights the value of multiperspective approaches in (participatory) research and SOD, to develop strategies for an inclusive educational environment through neurodiversity-informed decision processes and enable equitable learning environments for all students.

## 1. Introduction

The discourse on neurodiversity highlights an ever-existing dimension of heterogeneity in classrooms—the individual way that every brain and every nervous system processes information. People have always been neurodiverse (e.g., Singer, 1999; Walker, 2014; Chapman, 2020). In their review on the current discourse, McLennan et al. (2025) found “Neurodiversity is Human Diversity” as the core category that unifies all definitions. The term “Neurodiversity” primarily serves as a way to address this fact, while also advocating for systemic changes in order to develop an environment that caters to the needs of everyone (e.g., Grummt, 2023). This also hints at a distinction between a scientific and a political use of the term. Scientifically, “Neurodiversity” is mainly a descriptive concept to address a social phenomenon, based on neurological differences. As a political term, “Neurodiversity” is a normative call for action to address social injustice and discrimination based on neurological differences within an unaccommodating environment. While both perspectives share core attributes, these perspectives tend to mix; their distinction and different aims should be kept in mind. Following these thoughts, we will use the terms neurodivergent and neurotypical as sociological categories, as criteria in reference to mainstream society.

The development of an environment that caters to the needs of everyone is crucial when it comes to schools and their neurodiverse students. There are international legal obligations for public institutions—especially schools—to establish an environment that is suitable for the necessities of all students:

On the basis of the Right of Education for Everyone (Art. 28) in the UN-Convention on the Rights of the Child (United Nations, 1989), UN-Convention on the Rights of People with Disability (UN-CRPD) specifies this right by calling for “reasonable accommodations of the individual’s requirements” (United Nations, 2006, Art. 24e) for every child, regardless of individual ability. Furthermore, the 4-A Scheme states the obligation to make education available, accessible, acceptable and adaptable for everyone (Tomaševski, 2001). Finally, the fourth Sustainable Development Goal is to “ensure inclusive and equitable quality education […] for all” (United Nations, 2015, p. 14).

These are just some examples of documents that were developed on an international level to oblige countries and societies to support high-quality and equitable education for every child. As a consequence of this top-down principle, schools and teachers in every classroom are supposed to ensure participation, learning, and equity in education for each student.

Still, as we have stated before, schools have always been neurodiverse. This leads to the following question: why is it perceived as a “new” challenge in schools today?

The answer could be that the introduction of these paradigms—Neurodiversity and Mainstream Inclusive Education—necessitates a shift in the perception of the overall approach in the way schools have to care for children. Instead of a one-size-fits-all approach, schools must now cater to individual necessities, while also keeping a focus on the social and collective effects, to enable their students’ optimal individual and social development. As UN-CRPD puts it, “The full development of human potential and sense of dignity and self-worth, and the strengthening of respect for human rights, fundamental freedoms and human diversity” (United Nations, 2006, Art. 24a). This adds responsibilities for individual schools and leads to the need for support mechanisms to enable inclusive education, especially with respect to the heterogeneity of perception and learning in neurodiverse classes.

The project schAUT (full title: “Diagnose von Barrieren für autistische Schüler:innen in inklusiven Schulen”^1^) aimed to develop materials for schools to provide reasonable accommodations (in accordance with UN-CRPD, Art. 24) by diagnosing students’ individual barriers for education in schools on a collective level through a questionnaire and enabling *School Organizational Development* (SOD) towards greater barrier sensitivity. In short, schAUT’s aim is to reduce barriers to inclusive education in mainstream schools.

The German school system is highly segregated. Depending on the federal state, mainstream elementary schools last for four or six years. In the context of the project schAUT, it is important to note that the federal states North Rhine–Westphalia and Hesse have four years of elementary school, while Berlin has six. There are five different types of mainstream secondary schools—Hauptschule, Sekundarschule, Realschule, Gesamtschule, Gymnasium—which aim at different types of graduation. It is worth noting that all types are generally obliged to realize inclusive education, except for Gymnasium in some federal states (like North Rhine–Westphalia), which is aimed towards Abitur as graduation goal, the formal requirement for access to universities.

The project schAUT focused on barriers in education. Barrier, as a sociological term, addresses exclusion; its removal is a political act to grant accessibility to (social) spaces and situations (Trescher, 2022): “In this respect, drawing attention to barriers and demanding their removal constitutes an act of empowerment and self-authorization for specific individuals and groups threatened or affected by exclusion.” (Trescher, 2022, p. 460, translated) Additionally, our results show that barriers can be seen as a mirror to individual requirements, as developed for example in the Autistic-SPACE model by Doherty et al. (2023) (see Schwager et al., 2025 & Hümpfer-Gerhards et al., 2024a for more details). Our use of the term barrier addresses all types of reasons for social exclusion. This includes especially physical, social, and structural barriers.

Our research focuses on the subjective experience of barriers. Experiencing situations that result in overstimulation or mental overload hinders successful participation and learning (e.g., Waisman et al., 2022). They can become barriers in a gradual way or because of the cooccurrence of several of these influences. Asking for subjective experiences, in our opinion, is an effective way to address such hidden potential barriers. This way, we address the impact of all types of barriers on the individual, rather than trying to conceptualize an objective perspective on the occurrence of the sociological concept of barriers as an origin of exclusion.

Booth and Ainscow (2002) use barriers as a key factor in the *Index for Inclusion* to develop inclusive environments in mainstream schools. They pose central questions for SOD processes with regard to barriers (Booth & Ainscow, 2002, p. 40).

The term barriers therefore connects the concepts of neurodiversity, inclusion, and SOD and is thus of great value for a project like schAUT and this paper, for that matter. Our aim with the project’s products is to support schools in their efforts to create equity and reasonable accommodations in neurodiverse classrooms, in accordance with the concept of Universal Design for Learning (Biewer, 2023; Hall et al., 2012; Ralabate, 2011). This paper reports on data that was collected during the project. The provided analyses add to a previously published body of work from the project that reports in part on the same data (especially Fuhrmann & Schwager, 2021; Hümpfer-Gerhards et al., 2024a; Schwager et al., 2025).

The aim of this paper is to discuss crucial findings in the research process from data collected during the development and validation phases of the questionnaire, while also providing an overview of schAUT. We will focus on SOD processes in the context of inclusion and the lived reality of neurodiversity in the classroom, with a focus on (but not limited to) autistic students. We will provide insights into the following questions:

- Which barriers are the most important ones in mainstream schools?
- Are there significant differences in the subjective experience of barriers between neurodivergent and neurotypical students?
- How can barrier reduction be realized through SOD processes?

### Background on the Project schAUT

The project schAUT was funded by the German Federal Ministry of Education and Research (BMBF; FKZ: 01NV2104) and carried out by a participatory research group from Goethe University, Frankfurt a. M., Humboldt University of Berlin and White Unicorn e.V.

The main objective in the three-year project was to develop and validate a questionnaire for students in mainstream schools concerning their individual experience of barriers, while also researching ways to reduce barriers on a practical level.

Subjective experiences of autistic individuals served as a starting point for the project. During the research process, a more general perspective on the experience of neurodivergence and neurodiversity became the focal point. The questionnaire focuses on barriers for autistic people in a neurotypical environment, as it was based on a survey on this topic by Enthinderungsselbsthilfe (2008). However, every student in a class (or grade level) is supposed to fill it out anonymously. As an inclusive tool, the questionnaire is not supposed to single out the needs of individuals, but rather to provide an overview of possible barriers in an inclusive group. This approach is supported by the data, as every child is affected by some barriers, as we will show in Section 3.1. Thus, instead of individual feedback, an analysis tool shows a barrier profile on a group level and highlights the most impactful barriers.

In the questionnaire, we decided to ask for the general experience of barriers in the hypothetical case they appear, instead of asking for concrete situations that students experience in schools^2^. This way, the questionnaire can depict a more general profile of subjective barriers that can be used for schools to develop measures of barrier reduction, while also getting a sense of which barriers are the most urgent ones to target through SOD. Broadly speaking, if a school understands the general concept and urgency of a barrier, it will be able to act in advance, instead of just reacting to singular instances. This way, schAUT provides the necessary requirements for SOD.

Other products from the project schAUT include a manual on the practical reduction of specific barriers through SOD. It summarizes demands and ideas from the autistic community and school staff, which were collected during the research process. We also developed a program that helps schools in structuring SOD processes and the execution of the questionnaire. We will go into more detail on a prototypical process in Section 4.3.

All products from the project are free for use and can be accessed via the project website: www.schaut-verbund.de (accessed on 1 June 2026). 

The final questionnaires (for elementary and secondary schools) and the manual on the execution are provided in the Supplementary Files.

## 2. Materials and Methods

### 2.1. Participatory Research as a Foundation of the Project schAUT

SchAUT was carried out as a participatory research project. The collaboration between autistic and neurotypical researchers is an important aspect in the development of practical materials to prevent paternalistic attitudes and to value the importance of self-experience as part of the scientific process. While internationally this is more common in the field of autism research, as it proved to be of great importance for projects like LEANS (e.g., Alcorn et al., 2022) or Autistic-SPACE (e.g., Doherty et al., 2023), this was a great achievement in the German context. SchAUT was the first publicly funded educational research project where researchers with self-experience were paid equally to formally trained scientists.

In the process, we adhered to the principles for participatory research by von Unger (2014). “Participatory research is an umbrella term for research approaches that investigate and shape social reality in a collaborative, partnership-based manner. Its aim is both to understand and to transform social reality.” (von Unger, 2014, p. 1 translated) Following this definition, participatory research is defined by descriptive and transformative aims. This adds a layer of complexity, especially in a neurodiversity-based approach, with its double meaning of a scientific and political term as discussed above. Still, the general concept is research in the field of lived realities, which includes perspectives from affected individuals as collaborators in the process, as further elaborated by Keeley et al. (2019). von Unger (2014) describes participatory research in accordance with Bergold and Thomas (2010) not as a research methodology, but as a general style of research. To enact this style of research, we used Farin-Glattacker et al.’s (2014) grid for participatory research. We agreed on an equal collaboration for most steps of the formal process. This included planning and application for funds, conducting the project, and finally publication and implementation. The research needs were determined by White Unicorn e.V., which indicates an even higher grade of participation in Farin-Glattacker et al.’s (2014) scheme. The first idea for the project was proposed by members of White Unicorn e.V. It was refined in collaboration with colleagues from Humboldt University and Goethe University, which led to the collaborative draft of a research grant. After a positive review of the project and BMBF’s decision to fund it, equal participation became a focal point in the work process. In the beginning, a statute (schAUT, 2021) was agreed upon between the institutions to ensure collaboration and avoid mock participation (von Unger, 2014). Apart from an agreement to make key decisions through consensus, the conceptual direction of the project was laid out. This included the usage of a neurodiverse understanding of autism, the departure from paternalistic pedagogical approaches, and an empowering framework that embraces different perspectives. It also described providing reasonable accommodations as defined by UN-CRPD as the project’s focus (schAUT, 2021). The research process itself was characterized by collective approaches throughout all phases, with an emphasis on the expertise of both autistic and non-autistic researchers. White Unicorn e.V.’s connections to the German-speaking autistic community were especially valuable during the participants’ recruitment phase, as indicated by the sample sizes. Autistic and non-autistic researchers were involved in the development of research instruments and the process of data collection. All publications are authored by autistic and non-autistic researchers. This includes written publications, as well as conference appearances. The only point in Farin-Glattacker et al.’s (2014) grid without participation in the project was the reviewing and funding decision, at least to our knowledge. This was carried out by BMBF. Thus, no member of the research group influenced it.

To ensure equality, equal payment was an important step to reduce hierarchies between the co-researchers. Still, the participating institutions have uneven backgrounds. White Unicorn e.V. is an autistic self-advocacy group that specializes in scientific research on autism by founding a research institute. This leads to an uneven balance due to its smaller size in comparison to the two universities in the project, with their administrative background, reputation, and sheer size. To enable equal participation, we agreed on a veto right for every institution on every decision. This ensured that decisions could not be made by pure force of institutional background, ensuring a consensual approach to decision-making.

We reflected on our collaboration as a participatory research group throughout the whole project and developed principles for successful communication as part of the research process through ethnographic methods and analysis (Hümpfer-Gerhards et al., 2024b). As part of this process, we embraced occurring conflicts as a sign of real participation and an “indicator for the quality of participatory research” (von Unger, 2014, p. 85 translated).

The project schAUT was developed with regard to the principle of “Nothing about us, without us” (Charlton, 2004; Werner, 1998) and follows this principle in every phase of the process, to develop self-informed measures instead of descriptive outside perspectives, as is often the case in research on autism in education (Lindmeier, 2018). Therefore, we also agreed on avoiding clinical definitions of autism and deficit-oriented perspectives on neurodivergence. Instead, we adopted the definitions of neurodiversity (Walker, 2014) and autism (Walker, 2015) by Nick Walker. The use of the terms ‘autism’ and ‘autistic’ is also adopted as a self-description, as used by Walker (2015).

### 2.2. Data Collection and Materials

The project used a combination of quantitative and qualitative methods to investigate barriers and options for action. Throughout, there were quantitative online and paper-pencil surveys for potential barriers. Qualitative data were collected online from the autistic community and from school staff during workshops. Participants were students and staff from schools participating in the project schAUT and participants of a public online survey addressing the autistic community in Germany. Informed consent was obtained from all subjects involved in the studies. The study was approved by the Institutional Review Board of *Hessisches Ministerium für Kultus, Bildung und Chancen* (GWU 1113/12.05.2022).

For the quantitative analysis in this paper, we used data collected among students with the schAUT questionnaire. It measures the potential of specific situations to become a barrier for learning and social participation in everyday school life. Below, we briefly describe the stepwise development of the instrument.

The questionnaire is based on the results of three large online surveys, in sum collecting answers from more than 2000 autistic and non-autistic participants (see Schwager et al. (2025) for more details).

*Item generation and collecting qualitative suggestions for barrier reduction:* The first online survey was based on 27 typical barriers that occur for autistic adults (White Unicorn e.V. & Humboldt Universität Berlin, 2018). For each barrier, participants were asked to formulate typical situations in everyday school life from their experiences. Additionally, they were asked to formulate suggestions or solution strategies that could (have) improve(d) the specific situation in school for them. All answers to these open questions were subjected to qualitative content analysis (Mayring, 2010) in order to identify the situations that most frequently become barriers in everyday school life and to group and assign the suggested solution. During analysis, the number of barriers was reduced to 25 due to overlapping answers. Ultimately, four situations per barrier that were named most frequently served as the basis to generate items as brief descriptions of the situations.

*Validation and item reduction:* The Items were illustrated with child-friendly images. The potential subjective disruption caused by this situation could be scored on a five-point rating scale. There were two quantitative validation studies (online surveys). An additional purpose of these two studies was a sensible and well-founded reduction of the item number from 100 to 50 and a reliability test of the final item set and the five-point Likert scale.

*SchAUT questionnaire*: The quantitative data reported in this paper are based on a questionnaire that contains 25 potential barriers. Each barrier is represented by two items (see Table A1 in Appendix A) with the question “How much would this bother you?”. Participants should mark their subjective answer from 1 (It doesn’t bother me at all) to 5 (It bothers me so much, I can’t do anything anymore).

*Further qualitative data:* A staff workshop was held as part of the project at each participating school. The workshop contained feedback on the results of the questionnaire in their specific school. On this basis, the participating pedagogic staff (mostly teachers) were encouraged to collect and discuss realistic suggestions for barrier reduction in their school. Each workshop focused on two to three potential barriers per school, which were illustrated and underpinned by suggestions on barrier reduction from the autistic community. The discussions leading to decisions on the measures that will be implemented to reduce barriers were recorded and transcribed. Moreover, participants worked in small groups on their ideas and took systematic notes on flipcharts, which were also included in the qualitative evaluation.

### 2.3. Quantitative Analysis

*Data sample:* The quantitative data stem from a sample of 1092 students from 19 German mainstream schools in three federal states^3^ (9 primary schools, 10 secondary schools). The project focused on the first-year cohorts because the barriers can appear especially in new contexts. In all participating schools, at least one student in the cohort should be autistic or possibly autistic (according to parents’ information). As in Germany the transition to secondary school differs between federal states, there were 432 students from 1st, 292 students from 5th, and 368 students from 7th grades participating.

The reliability of the two-item barrier scales was tested through Spearman–Brown coefficients (Eisinga et al., 2013) because further analysis should be based on aggregated data. For the reliability tests, cases with missing data in one or both items were excluded. In the aggregated data, cases with one valid answer per barrier were kept while using this answer as an estimate for the missing ones. This case occurred rarely. The majority of missing data arose from accidental skipping of whole pages in the paper questionnaire. This is the cause of differing n’s in the analysis of barriers. The aggregated data per barrier will be visualized through violin plots (Hintze & Nelson, 1998) to show the range of the answers, while also indicating the general means. Our research interest lies in the relevance of the respective barriers and in the differences between neurotypical and neurodivergent students. Therefore, students who have been classified by their parents as autistic or possibly autistic are grouped together, as both groups appear to be more likely to experience barriers. Clinical criteria or proven psychiatric diagnoses are less relevant in this context. We will further discuss this approach in Section 4.1.

Means and range of potential disruption or distress, respectively, will be compared and discussed with regard to neurotypical and neurodivergent students.

### 2.4. Qualitative Analysis

*Data sample:* Autistic suggestions for barrier reduction were collected via the first online survey. A total of 699 people (mean age 26.7, SD 13.4) completed the online survey. There was no age restriction, because we wanted to collect a broad sample of subjective experiences that could also rely on reflections about one’s own past school days. A more distant point of view might enable additional ideas for barriers and potential solutions. Workshop data were collected from 17 schools^4^.

During the project, different types of qualitative data were collected. The aim of this paper is to systemize the collected data and set them in relation to each other as an illustrative example for SOD. All qualitative data were analyzed through qualitative content analysis (Mayring, 2010). The material was coded with deductive categories and sub-categories. The categories were filled via inductive coding from the data.

The barriers served as deductive categories for the analysis of the first online survey. For each category, the following deductive sub-categories were used^5^: “Possible solutions”, “stimming”, “coping”, “reactions”, “places in schools”, “situations”, and “not a problem”. For the data from the staff workshops, “assistance”, “statements on autistic students”, “obstacles and solutions”, and “general ideas for barrier reduction” served as deductive categories. For the last two, the barriers served as deductive sub-categories to sort the suggestions.

For the purpose of this paper, we focus on the sub-category “possible solutions” for data from the first online survey and the categories “obstacles and solutions” and “general ideas for barrier reduction” for data from the staff workshops. Three out of the twenty-five barriers (“Noise level”, “Mirrors and reflections”, and “Time pressure and urgency”) are chosen here for presentation of a detailed analysis of the suggestions in this paper. By doing so, we can establish illustrative links between the demands from the autistic community and SOD-based practical solutions. These barriers were chosen for their high-quality data throughout all phases, as we have records of detailed discussions on them during the workshops. Also, there is a direct connection between community demands (from the online survey) and the discussion during the workshops in schools. The intention of the analysis is to show prototypical ways of implementation. Thus, we chose examples with good documentation. However, this should not be generalized as an objective analysis for all processes, but rather as documentation of best practice.

For the analysis, we summarized demands and suggestions from the autistic community (from the first online survey) and discussions on the reduction of the barriers in schools (from the workshops on barrier reduction), as well as intended measures as a result of these discussions.

## 3. Results

### 3.1. Quantitative Analysis on Subjective Barrier Load

This section presents statistical results from the study. In the analysis, we focused on the reliability of the individual barriers and the distribution of the subjective barrier load, compared between neurotypical and neurodivergent participants.

Spearman–Brown coefficients for the barriers range from 0.34 to 0.85. As barriers are represented by just two items each, reliability is psychometrically sufficient on the level of single barriers for only 18 out of 25 barriers (see Table A1 in Appendix A). Usually, it is psychometrically not advantageous to form scales from just two items. So, seven of our barriers are not measured reliably here because of too much heterogeneity between the two answers per barrier. Satisfactory reliability can be achieved by summarizing more items of the schAUT questionnaire into larger barrier areas. We recommend this way of analysis when using the questionnaire (see Schwager et al. (2025) for more details). As our focus here lies on describing the perception of barriers and possible group differences on this level, we nonetheless stick with the presentation of data aggregated by barrier, knowing that this kind of analysis can be only exploratory at this point.

A comparison between neurotypical and neurodivergent participants might provide insight into the subjective experience of barriers in a neurodiverse group. The data stems from students in 19 different schools, so we view it as an example of distribution among children in schools in general. As the sample does not meet the criteria of representativity, the generalizability is limited. Rather, the results can serve as an insight into tendencies in neurodiverse schools. We chose to show the distribution in percentages instead of totals to provide comparability between the barriers and the two groups.

**Figure 1:**
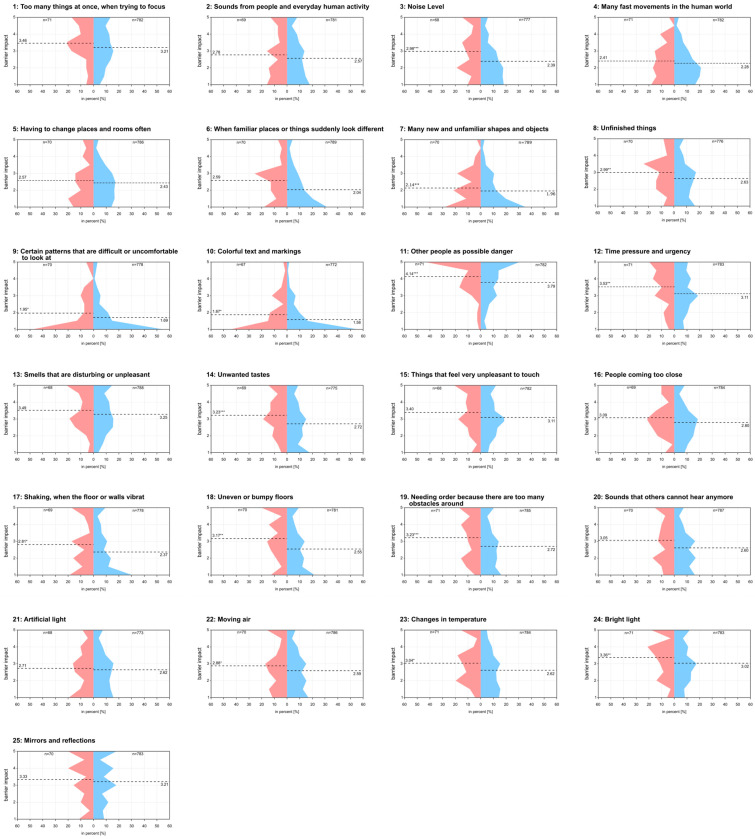
Violin plots of subjective barrier load between neurodivergent and neurotypical students. This figure shows a comparison between the subjective impact of each barrier for neurodivergent (red) and neurotypical (blue) participants. The two groups were selected by parents’ assessment/self-assessment. For each of the 25 barriers, participants were asked to assess two situations with the question “how much would this bother you?” using a scale between “It doesn’t bother me at all” (1) and “It bothers me so much, I can’t do anything anymore” (5). The different plots show the aggregated score for each barrier by percentage of participants (*Y*-axis). As there were two items per barrier, only .0 and .5 aggregated results were possible. This presentation gives detailed information on the data, as it provides insight into the different answers and highlights the diversity of the answers. The mean average for each group is indicated by the dotted line. Its value is stated for each group in every plot. The stars indicate significant results (*: *p* < 0.05; **: *p* < 0.01; ***: *p* < 0.001).

For all 25 barriers, a comparison of neurotypical and neurodivergent participants reveals higher mean levels of subjective barrier load for the neurodivergent group (Figure 1). This difference approaches statistical significance for 14 out of the 25 barriers. However, the answers in both groups are highly divergent. In every barrier and group, we found participants with the maximum and minimum ratings. This means that each barrier was rated as highly impactful, at least by some individual students in both groups. To conclude, while for all barriers the mean rating was higher in the group of neurodivergent participants, the depicted barriers were perceived as stressful for some individuals, regardless of neurodivergence, as we will discuss further in Section 4. Also, we observed the subjective impact of barriers for both groups, not just neurodivergent participants.

The barriers with the highest mean scores were: 11—*Other people as possible danger* (4.41 for neurodivergent and 3.79 for neurotypical participants), 1—*Too many things at once, when trying to focus* (3.46/3.21), 25—*Mirrors and reflections* (3.33/3.21), and 24—*Bright Light* (3.36/3.02). For barriers 1 and 25, the group difference does not approach significance, suggesting a rather similar intensity for neurodivergent and neurotypical students. This indicates that these barriers have the greatest negative impact on students and could be a focal point for SOD. However, as discussed above, the impact of a barrier is highly individual. It is possible—if not likely—that other smaller groups like different schools or classes should prioritize other barriers.

### 3.2. Qualitative Case Study on Measures in Barrier Reduction

**Figure 2:**
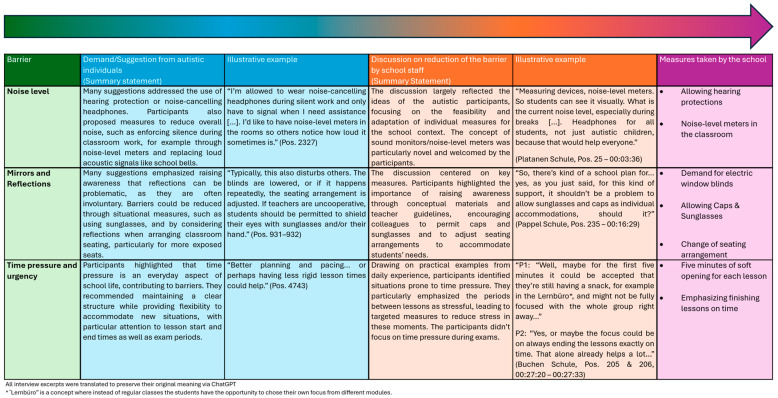
Measures of barrier reduction through SOD. The figure provides exemplary insights into the development of implemented measures through SOD to reduce three barriers. On the left side, the three barriers that are used as examples for this process are named. For each of the barriers, a summary statement as the result of qualitative analysis (Mayring, 2010) is introduced. This summarizes the main points of emphasis from the data that were collected in an online survey during the development process of the questionnaire from autistic participants. To provide further insight, an illustrative example from the data was chosen and translated to support the understanding of the demands. The blue columns represent data collected from the autistic community. The orange columns represent data from the workshops with school staff. This data was collected as part of the research process, when results from the questionnaire were presented to the schools. Suggestions for barrier reduction (summaries of the blue columns) were presented to the participants. The summary statement is once again a result of qualitative analysis (Mayring, 2010), and the (translated) illustrative example provides insight into the discussion. The column on the right finally shows measures to reduce the barriers that were agreed upon during the workshops. We can see a clear connection between the suggestions and the measures taken for each of the three examples. This shows examples for prototypical ways for SOD processes for these cases, where suggestions from (self-)experts inspire measures to reduce barriers.

In this section, we will provide insight into best-practice examples of SOD processes for barrier reduction in schools, with suggestions from the autistic community. As stated above (Section 2.4), as part of a workshop/group discussion, the participating school staff were introduced to suggestions for barrier reduction that were collected during the development of the material. This inspired concrete measures, as the following three exemplary means illustrate (Figure 2).

For all three examples, the suggested measures were adopted by the schools and incorporated into their individual settings.

For the barrier “Noise level”, the suggestions to allow hearing protection and noise-level meters were implemented as they were suggested. Interestingly, the latter was a novel suggestion for many participants from the school but was welcomed as a way of indicating noise to children in class. In this case, a suggestion from the autistic community provided new information to schools and became a point of emphasis in the discussion. The discussion among the school staff also revolved around the usability of headphones for all students, which indicates a neurodiverse and inclusive way of thinking, as both measures did not focus on autistic students in particular, but rather on the different demands and barriers for all students. This way, every student, including autistic individuals, can benefit from barrier reduction.

The second example focuses on the barrier “Mirrors and Reflections”. A point of emphasis in the suggestions was raising awareness for the general fact that this could be a barrier to learning, while also suggesting concrete measures to counter this barrier. During the discussion, the barrier was recognized as a problem for learning. The emphasis on raising awareness for this barrier during the discussion suggests that the staff agreed that this barrier is often overlooked in schools. This can be seen as an example where the introduction of autistic perspectives supported schools in reflecting on their perspectives. The measures were once again adapted, as they were seen as easy to implement. This school also used the understanding of reflections as a barrier to demand electric window blinds. The building is owned by the city, so the school cannot implement this measure by itself, but can use the results of the questionnaire as an argument for its demand.

Finally, the third example revolves around the barrier “Time pressure and urgency”. The suggestions concerned time flexibility to accommodate new situations while also staying on schedule in general, to provide a clear structure. These seemingly contradictory suggestions were an opportunity to reflect on scheduling in the school in general during the discussion. On the one hand, the school decided to emphasize finishing the lessons on time in order to reduce time pressure during breaks. On the other hand, they decided that lessons should start with a soft opening of five minutes. This provides flexibility in the schedule and reduces timing issues. This can be seen as an example where the suggestions were taken as a starting point and applied to existing structures. In this case, the expertise of the autistic community was recognized and supported the expertise of the school staff (in their particular environment) to find fitting ways to reduce the barrier.

## 4. Discussion

### 4.1. Subjective Barriers as an Indicator for Neurodiversity

In the first part of this discussion, we will focus on the implications of the quantitative results for a broader understanding of neurodiversity. We argue that the results support the general concept of neurodiversity as a perspective that highlights the subjectiveness of experiences and its practical implications as a natural form of human variation, in accordance with Larrauri et al.’s (2025) “Big-Tent” approach.

First, we provide an explanation of our grouping strategy: We discussed extensively whether to ask students for a formal diagnosis. One major concern, particularly raised by our autistic project partner, was that relying on diagnosis reproduces a clinical and deficit-oriented perspective on autism. It also creates barriers for students or parents who do not want to disclose an autism diagnosis or avoid formal assessment altogether because of stigma or privacy concerns. In addition, access to diagnostic services is often limited, meaning that some autistic students may remain undiagnosed and would therefore be excluded from the study. Self-identification, by contrast, is more inclusive and allows for greater anonymity. However, it also introduces methodological limitations, since self-reported “possible autism” may include a wide range of characteristics that are not necessarily specific to autism. As a result, there is a risk that the group includes not only students with autistic traits, which may increase heterogeneity and potentially dilute the findings. Nevertheless, we would argue, if the focus is understood more broadly as neurodivergence rather than narrowly defined autism alone, this approach remains appropriate. Moreover, despite these methodological risks, the observed differences are still very pronounced.

When comparing the mean subjective impact of barriers, we found a higher impact for the group of neurodivergent participants for all barriers in comparison to the group of neurotypical participants (Figure 1). Though not all differences are significant, we still argue that this could suggest that neurodivergent students are at risk of facing more situations that qualify as barriers in schools, as they are generally more impacted by them. When talking from a normative perspective on neurodiversity, this indicates that schools might be generally less suited for the needs of autistic and neurodivergent students and are more likely to fail in providing “reasonable accommodations”—as UN-CRPD puts it—for this group of students. This result could be seen as an implication that there is value in a finite distinction between neurodivergent and neurotypical students, especially when it comes to practical implications. However, when looking at the results in more detail, we oppose this interpretation. As described above, the results show great diversity in subjective experience. For every barrier, we saw some participants from both groups with the highest possible impact rating (Figure 1). This indicates that students can be affected by barriers, regardless of neurodivergence. We therefore argue that the higher mean subjective impact for neurodivergent students should be interpreted carefully as a higher likelihood that these students might be impacted by the barriers in this study. Thus, we suggest an inclusive approach that emphasizes neurodivergent students’ experiences while taking the experiences of all students into account.

In their comment in Nature Mental Health, Larrauri et al. (2025) argue for an understanding of neurodiversity that emphasizes diversity of perception and individual experience:

*“Neurodiversity is a concept that acknowledges the wide range of neurological differences in how the brain functions, encompassing variations in learning, attention, social interaction and sensory processing, among other skills and traits. These differences are rooted in biology, and, along with the multifaceted impact of various societal and environmental factors, result in individuals perceiving and interacting uniquely with the world.”*

(Larrauri et al., 2025, p. 271)

This understanding of the term aligns with common definitions like Chapman’s (2020), Singer’s (1999), Walker’s (2014), or Kapp et al.’s (2013). In a scoping review on the scientific conceptualization of neurodiversity, McLennan and colleagues found “Neurodiversity is Human Diversity” as the central category, which unifies all approaches towards the topic (McLennan et al., 2025), although some authors like May (2025) criticize this understanding as negating differences instead of embracing them.

General differences in subjective experience between neurotypical and neurodivergent groups were also found in empirical approaches, for example by McLeod et al. (2019), Isenstein et al. (2021), or von Hausen et al. (2025). Our study presents another example of empirical evidence for the subjectiveness of lived experience between neurotypical and neurodivergent groups, especially in schools. According to Larrauri et al.’s (2025) “Big-Tent” approach, this should be seen as natural variation under the “Big Tent” of human experience. In their suggestion for *Prototypical Autism*, Mottron and Gagnon (2023) suggest an understanding of autism as an asymmetric bifurcation in early childhood development. Taking autism as an example for neurodivergence, this normalizes biological differences as part of natural variation, which is just statistically less common, like twin pregnancy or left-handedness.

This leads to the question of implications from this approach:

*“Because certain traits are more common in the general population, the world is predominantly structured around the needs of ‘neurotypical’ individuals who share similar cognitive and behavioral profiles.”*

(Larrauri et al., 2025, p. 271)

This perspective helps in the understanding of our results on barriers in schools. We found that the average impact of the tested barriers is higher for our neurodivergent participants, which makes sense when we take a look at the selection of the barriers in our study. We started with a selection of barriers that autistic people experience in society in general (Enthinderungsselbsthilfe, 2008). Thus, our selection was biased towards barriers for autistic students in a world that is historically designed towards a neuro-normative standard (Larrauri et al., 2025, p. 272). Accordingly, we focused on situations that were more likely to be perceived as barriers for neurodivergent participants, hence the overall higher subjective barrier load. It might be interesting to use the same approach as we did, but focus on hypothetical barriers for neurotypical students. Depending on the barrier, which might not be present due to the neuro-normative standard design of schools, we would assume that the results may show equal differences between the groups. Only the barrier load might be higher for the group of neurotypical participants—which could further support the “Big-Tent” approach.

However, when discussing practical implications of our results, assuming the world is currently designed towards the needs of an assumed neuro-normative standard profile (Larrauri et al., 2025), in accordance with our results, we would argue that it still makes sense to focus on barriers for neurodivergent groups, while also taking the heterogeneity of subjective barrier impact into account as part of the “Big-Tent” of human experiences.

Our results also mirror Doherty et al.’s (2023) concept of Autistic SPACE. The 25 barriers identified in the project schAUT correspond closely to the needs of autistic individuals as conceptualized in the Autistic-SPACE model by Doherty et al. (2023), particularly in terms of physical, cognitive, and emotional space. These barriers can thus be understood as concrete manifestations of unmet needs within these dimensions. Furthermore, the project’s recommendations for barrier reduction—derived both from the autistic community and from school-based workshops—can be systematically aligned with the SPACE framework. They include sensory adaptations (sensory), announcements, transparency, and structure to mitigate surprises and time pressure (predictability), as well as consideration and mutual understanding (acceptance, communication, empathy).^6^

Our results indicate that the schAUT program could be useful for an informed approach towards SOD processes, in particular and policy decisions that shape society and environment in general. We also argue that our results support both descriptive and normative perspectives on neurodiversity.

### 4.2. Barrier Reduction as an Assignment for School Organizational Development (SOD)

Rolff (2018) identifies organizational, personal, and teaching development as the main areas of SOD. While not strictly separated, each barrier in schAUT can be associated with at least one of Rolff’s areas. According to this, we are confident that the developed material can be useful in providing important information for SOD processes.

Wicki et al. (2025) add governance as another crucial dimension for SOD. As schAUT’s design is targeted at the school level, the barriers generally do not aim explicitly at the area of governance. Still, “[…] educational change is not a linear top-down process but a negotiated adaptation involving actors at multiple levels” (Wicki et al., 2025, p. 4). The results of the questionnaire can be used as proof of necessity with authorities, when it comes to cost allocation—as one school did in their request for electric window blinds (Figure 2).

Barriers are a key factor for the development of inclusive mainstream schools. The term itself addresses exclusion, while their removal can be seen as an act of empowerment (Trescher, 2022). Booth and Ainscow (2002) used the identification of barriers as a category in their *Index for Inclusion* (p. 40). The transformative aim of the project schAUT is to support the development of barrier-reduced learning environments for all students in the sense of a Universal Design for Learning (e.g., Biewer, 2023; Hall et al., 2012; Ralabate, 2011).

A point of emphasis in the development was providing different perspectives on barriers to support SOD-processes. This proved to be beneficial in the documented processes. The suggestions from the autistic community for the reduction of barriers inspired practical ideas for concrete measures, as they added another layer of expertise and insight into the perceived impact of barriers. Participants described that some of the ideas were new to them and added new ways of thinking (Figure 2). A main objective of SOD is to find effective measures and fitting solutions for the individual school, as there is no one-size-fits-all approach in the development of inclusive settings (e.g., Wicki et al., 2025). The incorporation of autistic perspectives on barrier reduction proved to be a great resource and benefit in the process.

As the questionnaire serves as the starting point of schAUT, students’ perspectives become the focal point in the identification of crucial barriers in schools, thus highlighting areas of development for SOD processes. This aligns with Gasteiger-Klicpera and Paleczek’s (2025) research on the Inclusive Inquiry Approach (Messiou & Ainscow, 2020). The study found positive effects of multiple perspectives in the exemplary development processes. Although the approach is more nuanced and focuses on the teaching dimension (Rolff, 2018), the results still suggest that SOD benefits from multi-perspectivity, especially students’ perspectives.

Finally, we saw incidents where the material supported the understanding of neurodiversity in general and thus influenced the attitudes of school staff. Generally speaking, attitudes towards inclusion are a deciding factor when it comes to inclusion in general (e.g., Avramidis & Norwich, 2002; Beacham & Rouse, 2012), and SOD processes in particular (e.g., Bengel, 2021; Wächter & Gorges, 2023; Wicki et al., 2025).

### 4.3. A Prototypical SOD Process Through schAUT

Finally, we will provide insight into the use of schAUT as a program for SOD through a prototypical description of the use of the developed material. This is obviously a transformative perspective in the sense of a normative usage of the term neurodiversity.

SchAUT encourages schools to use it autonomously as an in-house workshop. All materials are free to use and accessible via an online resource^7^. It is recommended that a group of school staff take responsibility for preparing workshops. In providing the material, we encourage these staffers to focus on important aspects for their school in particular. We provide readymade videos for the workshops to reduce the workload for preparation. The first workshop takes about one hour and focuses on the concepts of SOD, neurodiversity, and autism in general. It also provides information on the execution of the questionnaire, which is the second step of the program. We provided an analysis tool for the schools for easy evaluation of the results from the questionnaire. This highlights crucial barriers automatically. Based on the results, we suggest another workshop where the results of the questionnaire can be discussed. Each barrier is explained theoretically and in the form of narrative videos to provide insight into the subjective experiences. This is also the point where the suggestions from the autistic community are provided to inspire measures as part of a presentation specific to each barrier. The workshop continues with an evaluation of the state of the school in the matter of developing a neurodiversity-friendly environment. To support this process, we also developed an evaluation grid based on Landwehr and Obrist (2012) to identify general fields of improvement. The workshop finishes with the suggestion to agree on measures the school wants to take to reduce crucial barriers. The final step of the program is an evaluation of the measures, after a testing phase and the opportunity to agree on further steps.

Although extensive, the program is developed adaptively to support schools without demanding too much of the limited time resources that school staff often tend to have. It is also accompanied by a manual that goes into further detail to support the processes and provides even more information if necessary. We are confident that this setup supports SOD processes. An evaluation study is currently being conducted, and preliminary results seem promising regarding the use of the questionnaires and the manual.

## 5. Conclusions

The project schAUT provides an insight into the experiences of barriers in a neurodiverse group of students. We are positive that this approach can be used to identify measures for a neurodiversity-informed approach to SOD, pending the final results of the evaluation study. The developed material can be used to reduce barriers without singling out individual students; instead, they provide reasonable accommodations for a neurodiverse group of students—as all students are at risk of experiencing barriers. This supports the development of schools as a neurodiversity-friendly environment and enables the principles of a *Universal Design for Learning* (Biewer, 2023; Ralabate, 2011). A heterogeneity of perspectives can be beneficial for SOD processes. The schAUT program uses the experience of individual students as the focal point of barrier reduction and provides deeper information on experience and ideas of barrier reduction from the autistic community, to inspire concrete measures in schools.

On a theoretical level, our data supports the general concept of neurodiversity, as it highlights the subjectiveness of barriers. We argue that barrier reduction, as a SOD process, should focus on neurodivergent experiences, as they are often more affected by barriers, but take all perspectives into account. Barrier reduction can be beneficial for everyone, regardless of neurodivergence. The results support the concept of neurodiversity as a way to address social exclusion.

## Data Availability

For further information on the project, and a full list of publications from the project, please consider https://forschung-inklusive-bildung.de/schulische-bildung/schaut/ (accessed on 1 June 2026). Data publication is currently being executed and will be linked there as well.

## Notes

1 Transl.: “Diagnostic of barriers for autistic students in mainstream schools”.

2 The items consist of short descriptions of situations (in the form of text and pictures) with the question “How much would this bother you?” Participants can answer on a 5-point Likert scale between “It doesn’t bother me at all” (1) and “It bothers me so much, I can’t do anything anymore” (5).

3 Berlin, Hesse, and North Rhine–Westphalia.

4 In one school, a participant denied their consent to further research analysis. Accordingly, the data was excluded from analysis, and one school did not find a possibility to take the workshop within the project period because of organizational restructuring.

5 The analysis was conducted in German. For a better understanding, we translated the (sub-)categories for this paper.

6 Please refer to Hümpfer-Gerhards et al. (2024a), Chapter 7, for further insights on this.

7 https://hu.berlin/schAUT (accessed on 1 June 2026).

## Supplementary Materials

The following supporting information can be downloaded at https://www.mdpi.com/article/10.3390/bs16060949/s1. For further information and download of all the developed materials, please refer to www.schaut-verbund.de (accessed on 15 April 2026).

## Abbreviations

The following abbreviations are used in this manuscript:

BMBF	German Federal Ministry of Education and Research
SDGs	Sustainable Development Goals
SOD	School Organizational Development
UN-CRPD	United Nations Conventions on the rights of Persons with Disabilities

## Appendix A

**Table A1:** Barriers, items, sample size/barrier and Spearman–Brown coefficient/barrier.

	Barrier Description	Items	n Included	Spearman-Brown- Coefficient *
1	Too many things at once, when trying to focus	1a. I want to listen in class, but the others are chatting. 1b. Someone interrupts me in the middle of a task and wants something from me.	1057	0.682
2	Sounds from people and everyday human activity	2a. The clicking of shoes echoes through the whole hallway. 2b. I can hear the music lesson from the room next door.	1067	0.638
3	Noise Level	3a. During breaks, it is very noisy. 3b. The classroom is quiet, but some classmates are whispering loud enough that I can hear them.	1061	*0.591*
4	Many fast movements in the human world	4a. There is a lot of rushing and moving in the hallway and on the stairs. 4b. On the way to school, there are many people and vehicles around.	1065	*0.566*
5	Having to change places and rooms often	5a. In the new room, a different classmate is sitting next to me. 5b. We change rooms a lot, and I have to find the right one.	1069	*0.414*
6	When familiar places or things suddenly look different	6a. The classroom walls have been painted in a different color. 6b. The tables were moved, and suddenly everyone is sitting somewhere else.	1075	*0.582*
7	Many new and unfamiliar shapes and objects	7a. The teacher looks different than usual. 7b. Today we are working with new things that I do not know at all.	1074	*0.522*
8	Unfinished things	8a. My picture is not finished yet, but I have to stop painting because art class is over. 8b. The topic of the lesson is not finished, but we still change to another subject.	1062	0.608
9	Certain patterns that are difficult or uncomfortable to look at	9a. There are lots of different patterns on my notebook. 9b. There are many small patterns on the classroom floor.	1055	0.679
10	Coloured text and markings	10a. Everything on the board is written in different colors. 10b. The poster in the classroom has very bright colors.	1051	0.597
11	Other people as possible danger	11a. Some teachers are rough and do not want to help me. 11b. Some children insult me, do not let me join in, or threaten me.	1068	0.689
12	Time pressure and urgency	12a. After swimming, I have to dry myself and get dressed quickly because the bus is waiting. 12b. The teacher calls on me without warning, and I cannot think first.	1068	*0.582*
13	Smells that are disturbing or unpleasant	13a. I go into the school bathroom, and it smells bad. 13b. I can smell the teacher’s perfume during class.	1076	*0.561*
14	Unwanted tastes	14a. At school, everyone gets the same lunch, and I do not like the taste. 14b. The same food tastes different every time.	1051	0.634
15	Things that feel very unpleasant to touch	15a. When clearing the table, I have to touch dirty napkins and leftover food. 15b. In sports class, the teams get plastic bibs that feel strange.	1062	*0.569*
16	People coming too close	16a. During quiet work time, the teacher puts her hand on my arm and explains the task again in my notebook. 16b. In the stairway, it is often very crowded, and other children are too close to me.	1065	*0.337*
17	Shaking, when the floor or walls vibrate	17a. When everyone runs out for break, the whole building shakes. 17b. The tables in the classroom sometimes shake, because there is a truck outside.	1059	0.784
18	Uneven or bumpy floors	18a. The floor tiles are old and broken. 18b. The schoolyard is uneven and has holes.	1070	0.784
19	Needing order because there are too many obstacles around	19a. Someone does not put something back where it belongs. 19b. The wardrobe in the classroom is very messy	1073	0.652
20	Sounds that others cannot hear anymore	20a. The lamps in the classroom are buzzing. 20b. The teacher’s computer is humming and making high sounds.	1068	0.766
21	Artificial light	21a. The light from the lamp feels cold. 21b. The lamps in the classroom are flickering.	1056	*0.568*
22	Moving air	22a. When the classroom is aired out, there is a draft. 22b. My hair blows into my face when it is windy outside or when air comes through the open window.	1065	0.615
23	Changes in temperature	23a. It gets cold when the room is aired during class. 23b. There are different in temperatures everywhere—in the classroom, in the hallway, and in the schoolyard.	1065	0.612
24	Bright light	24a. Sunlight shines directly on me from outside. 24b. The room light shines straight into my face.	1063	0.6
25	Mirrors and reflections	25a. Sunlight reflects in the clock and dazzles me. 25b. The sun dazzles me when it reflects off the building across from us.	1068	0.845

* insufficient reliability coefficients in italics.

## References

[B1-behavsci-16-00949] Alcorn A. M., Fletcher-Watson S., McGeown S., Murray F., Aitken D., Peacock L. J. J., Mandy W. (2022). Learning about neurodiversity at school: A resource pack for primary school teachers and pupils.

[B2-behavsci-16-00949] Avramidis E., Norwich B. (2002). Teachers’ attitudes towards integration/inclusion: A review of the literature. European Journal of Special Needs Education.

[B3-behavsci-16-00949] Beacham N., Rouse M. (2012). Student teachers’ attitudes and beliefs about inclusion and inclusive practice. Journal of Research in Special Educational Needs.

[B4-behavsci-16-00949] Bengel A. (2021). Schulentwicklung inklusion: Empirische einzelfallstudie eines schulentwicklungsprozesses.

[B5-behavsci-16-00949] Bergold J., Thomas S., Mey G., Mruck K. (2010). Partizipative Forschung. Handbuch qualitative forschung in der psychologie.

[B6-behavsci-16-00949] Biewer G., Frohn J., Bengel A., Piezunka A., Simon T., Dietze T. (2023). Universal design for learning (UDL) als entwicklungsperspektive für einen inklusiven Unterricht. Inklusionsorientierte schulentwicklung: Interdisziplinäre rückblicke, einblicke und ausblicke.

[B7-behavsci-16-00949] Booth T., Ainscow M. (2002). Index for inclusion: Developing learning and participation in schools.

[B8-behavsci-16-00949] Chapman R., Bertilsdotter Rosqvist H., Chown N., Stenning A. (2020). Defining neurodiversity for research and practice. Routledge advances in sociology. Neurodiversity studies: A new critical paradigm.

[B9-behavsci-16-00949] Charlton J. I. (2004). Nothing about us without us: Disability oppression and empowerment.

[B10-behavsci-16-00949] Doherty M., McCowan S., Shaw S. C. (2023). Autistic SPACE: A novel framework for meeting the needs of autistic people in healthcare settings. British Journal of Hospital Medicine.

[B11-behavsci-16-00949] Eisinga R., Grotenhuis M., Pelzer B. (2013). The reliability of a two-item scale: Pearson, Cronbach, or Spearman-Brown?. International Journal of Public Health.

[B12-behavsci-16-00949] Enthinderungsselbsthilfe (2008). Grundzüge der kollision autistischer eigenschaften mit nichtautistisch geprägter umgebung.

[B13-behavsci-16-00949] Farin-Glattacker E., Kirsching S., Meyer T., Buschmann-Steinhage R. (2014). Partizipation an der Forschung—Eine Matrix zur Orientierung.

[B14-behavsci-16-00949] Fuhrmann S., Schwager S. (2021). Umfrage zu Barrieren für Autisten: Entwicklung eines Online-Fragebogens.

[B15-behavsci-16-00949] Gasteiger-Klicpera B., Paleczek L. (2025). Enhancing inclusion in the education system through school and lesson development. The concept of inclusive inquiry. Bildung Für Alle Stärken. Ein Handbuch Für Die Evidenzbasierte Entwicklung Inklusiver Schulen.

[B16-behavsci-16-00949] Grummt M., Lindmeier C., Grummt M., Richter M. (2023). Einführung in das paradigma der neurodiversität. Neurodiversität und Autismus.

[B17-behavsci-16-00949] Hall T. E., Meyer A., Rose D. H. (2012). What works for special-needs learners. Universal design for learning in the classroom: Practical applications.

[B18-behavsci-16-00949] Hintze J. L., Nelson R. D. (1998). Violin plots: A box plot-density trace synergism. The American Statistician.

[B19-behavsci-16-00949] Hümpfer-Gerhards L., Fuhrmann S., Schwager S., Kleres J., Kunert J., Benecke M., Knigge M., Moser V. (2024a). Schule & Autismus—schAUT: Barrieresensible Gestaltung inklusiver Schulen *(Eine Handreichung)*.

[B20-behavsci-16-00949] Hümpfer-Gerhards L., Kunert J., Fuhrmann S., Hartwieg S., Moser V., Knigge M., Benecke M., Schwager S. (2024b). Erwartungsmanagement als schlüssel für partizipative forschung: Kritische reflexion in theorie, empirie und erleben aus dem forschungsprojekt schule und autismus (schAUT). Gemeinsam Leben.

[B21-behavsci-16-00949] Isenstein E. L., Park W. J., Tadin D. (2021). Atypical and inflexible visual encoding in autism spectrum disorder. PLoS Biology.

[B22-behavsci-16-00949] Kapp S. K., Gillespie-Lynch K., Sherman L. E., Hutman T. (2013). Deficit, difference, or both? Autism and neurodiversity. Developmental Psychology.

[B23-behavsci-16-00949] Keeley C., Munde V., Schowalter R., Seifert M., Tillmann V., Wiegering R. (2019). Partizipativ forschen mit Menschen mit komplexem Unterstützungsbedarf. Teilhabe.

[B24-behavsci-16-00949] Landwehr N., Obrist M. (2012). Instrumente zur Schulevaluation und zur Schulentwicklung Bewertungsraster zu den schulischen Integrationsprozessen an der Aargauer und der Solothurner Volksschule.

[B25-behavsci-16-00949] Larrauri C. A., Stein M. A., Wickramasinghe A., Ntolkeras G., Yassin W. (2025). Neurodiversity-informed inclusive under-standing of neurological differences. Nature Mental Health.

[B26-behavsci-16-00949] Lindmeier C. (2018). Kinder und Jugendliche aus dem Autismus-Spektrum in der Schule. Forschungsfelder und Forschungsdesiderate. Zeitschrift Für Heilpädagogik.

[B27-behavsci-16-00949] May J. (2025). Neurodiversity with Nuance. Neuroethics.

[B28-behavsci-16-00949] Mayring P. (2010). Qualitative inhaltsanalyse: Grundlagen und techniken.

[B29-behavsci-16-00949] McLennan H., Aberdein R., Saggers B., Gillett-Swan J. (2025). Thirty years on from sinclair: A scoping review of neurodiversity definitions and conceptualisations in empirical research. Review Journal of Autism and Developmental Disorders.

[B30-behavsci-16-00949] McLeod J. D., Meanwell E., Hawbaker A. (2019). The experiences of college students on the autism spectrum: A comparison to their neurotypical peers. Journal of Autism and Developmental Disorders.

[B31-behavsci-16-00949] Messiou K., Ainscow M. (2020). Inclusive Inquiry: Student–teacher dialogue as a means of promoting inclusion in schools. British Educational Research Journal.

[B32-behavsci-16-00949] Mottron L., Gagnon D. (2023). Prototypical autism: New diagnostic criteria and asymmetrical bifurcation model. Acta Psychologica.

[B33-behavsci-16-00949] Ralabate P. K. (2011). Universal Design for Learning: Meeting the Needs of All Students. The ASHA Leader.

[B34-behavsci-16-00949] Rolff H.-G. (2018). Schulentwicklung kompakt: Modelle, instrumente, perspektiven (Neu ausgestattete Sonderausgabe, 3., vollständig überarbeitete und erweiterte Auflage). Neue basis-bibliothek schulleitung.

[B35-behavsci-16-00949] schAUT (2021). Projekt-Statut schAUT.

[B36-behavsci-16-00949] Schwager S., Hümpfer-Gerhards L., Kunert J., Fuhrmann S., Benecke M., Knigge M., Moser V., Beck K., Ferdigg R. A., Katzenbach D., Kett-Hauser J., Laux S., Urban M. (2025). Barrierendiagnostik und die Entwicklung eines barrieresensiblen Umfelds als Grundlage inklusiver Schulentwicklung mit besonderem Blick auf autistische Schüler:innen—Ergebnisse des schAUT-projekts. Förderbezogene diagnostik in der inklusiven bildung. Professionalisierung—Spezifische unterstützungsangebote—Übergänge in die berufliche Bildung.

[B37-behavsci-16-00949] Singer J., Corker M., French S. (1999). ‘Why can’t you be normal for once in your life?’: From a ‘problem with no name’ to the emergence of a new category of difference. Disability, human rights and society. Disability discourse.

[B38-behavsci-16-00949] Tomaševski K. (2001). Human rights obligations: Making education available, accessible, acceptable and adaptable.

[B39-behavsci-16-00949] Trescher H., Kessl F., Reutlinger C. (2022). Barriere. Sozialraumforschung und Sozialraumarbeit. Sozialraum.

[B40-behavsci-16-00949] United Nations (1989). Convention on the rights of the child.

[B41-behavsci-16-00949] United Nations (2006). Convention on the rights of persons with disabilities and optional protocol.

[B42-behavsci-16-00949] United Nations (2015). Transforming our world: The 2030 agenda for sustainable development: Resolution adopted by the general assembly on 25 September 2015 *[A/Res/70/1]*.

[B43-behavsci-16-00949] von Hausen F., Larraín-Valenzuela M. J., Carcamo B., Salgado-Obregon N. (2025). Autistic and neurotypical variance in the appraisal of emotional and interoceptive words. Frontiers in Communication.

[B44-behavsci-16-00949] von Unger H. (2014). Partizipative forschung: Einführung in die forschungspraxis. Lehrbuch.

[B45-behavsci-16-00949] Waisman T. C., Alba L. A., Green S. A. (2022). Barriers to inclusive learning for autistic individuals. Pediatrics.

[B46-behavsci-16-00949] Walker N. (2014). Neurodiversity: Some basic terms & definitions.

[B47-behavsci-16-00949] Walker N., Sutton M. (2015). What is Autism?. The real experts: Readings for parents of autistic children.

[B48-behavsci-16-00949] Wächter T., Gorges J. (2023). Wie kommt Inklusion in die Schulen? Einstellung zur Inklusion als Prädiktor der inklusionsbezogenen Fortbildungsmotivation von Lehrkräften. Zeitschrift Für Erziehungswissenschaft.

[B49-behavsci-16-00949] Werner D. (1998). Nothing about us without us: Developing innovative technologies for, by and with disabled persons/David Werner; with the PROJIMO team and many friends; drawings and photos by the author.

[B50-behavsci-16-00949] White Unicorn e.V., Humboldt Universität Berlin (2018). Umfrage zu Barrieren für Autisten. Entwicklung eines Online-Fragebogens.

[B51-behavsci-16-00949] Wicki M. T., Törmänen M., Altmeyer S., Carpelan R., Erzinger A. B., Gasteiger-Klicpera B., Gebhardt M., Koenig O., Knigge M., Löser J., Merz-Atalik K., Moser V., Paju B., Rabenstein K., Savolainen H., Schurig M. (2025). Evidence-based development of inclusive schools—A review of European research. International Journal of Inclusive Education.

