# Supplementary material for: Reducing Barriers in Neurodiverse Schools—schAUT: A Program to Identify and Reduce Barriers for Autistic and All Students
Source: Behav Sci (Basel). 2026 Jun 9;16(6):949. doi: 10.3390/bs16060949 (PMC13296199; doi:10.3390/bs16060949)
Supplement: Supplementary file 1 [file behavsci-16-00949-s001.zip › Supplementary FIles/Manual.pdf]

Der schAUT-Barrierenfragebogen

Manual

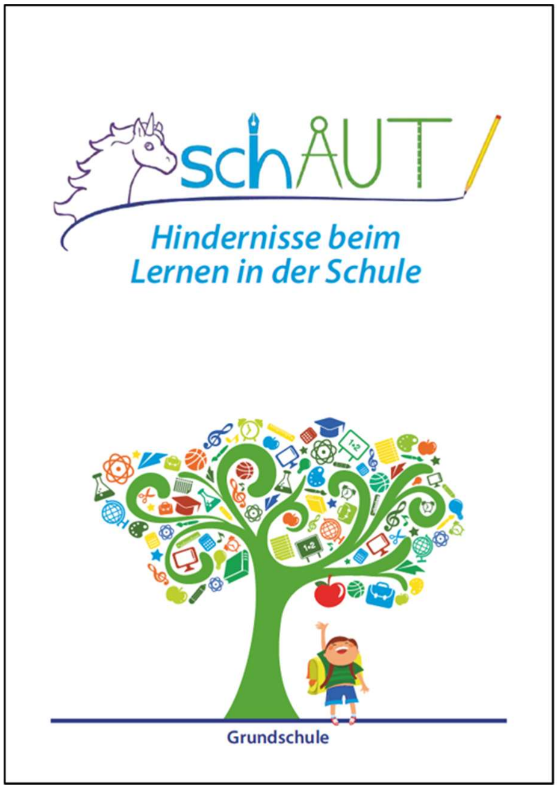

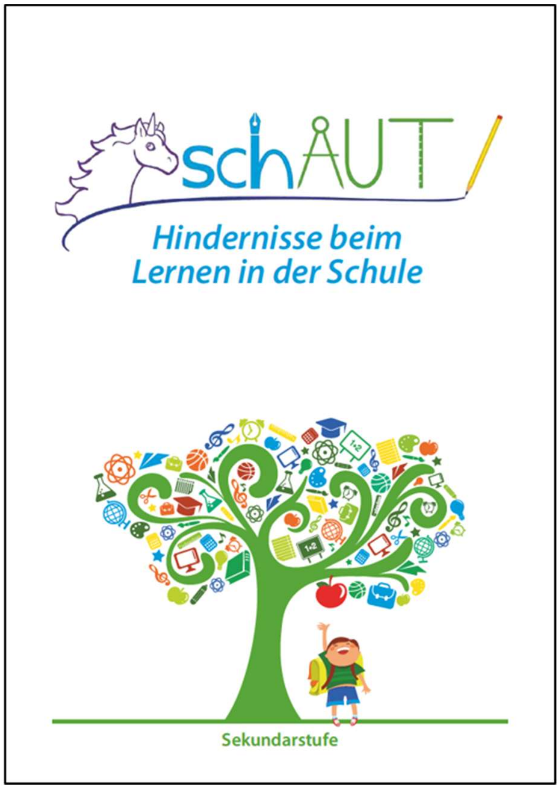

Inhalt

Einführung 1
Befragung der Schüler:innen mit dem schAUT-Barrierenfragebogen 2
  Umfeld für die Befragung 2
  Anonymisierung 2
  Anleitung 2
Auswertung des schAUT-Barrierenfragebogens 3
  Eingabe der Daten 3
  Informationen aus den Auswertungen 4
  Nutzung der Ergebnisse für die Arbeit mit der Handreichung 6
Entwicklung des Instruments und Güte des Barrierenfragebogens 7
  Welche Barrieren für Autist:innen gibt es? 7
  Von den Barrieren zu Fragebogenitems 7
  Von 100 Fragebogenitems zu 50 Fragebogenitems und acht Barrierenbereichen 8
  Wissenschaftliche Gütekriterien für die finale Fragebogenversion 9
Quellen 10
Materialien 11
  Durchführungsanweisungen Grundschule 11
  Durchführungsanweisungen Sekundarstufe 15
  Demonstrationsvorlage Skala 18
  Vorlage Elternbrief 21

Einführung

Mit dem schAUT-Barrierenfragebogen kann für verschiedene Bereiche erfasst werden, wie sehr bestimmte Reize oder Situationen Barrieren für Schüler:innen darstellen können. So wird es ermöglicht, die Bedarfe innerhalb Ihrer Klasse besser zu erkennen und ggf. auf die Vermeidung bestimmter Barrieren hinzuarbeiten. Die Befragung mit dem schAUT-Barrierenfragebogen kann ebenfalls die Grundlage dafür bilden, mit den Schüler:innen über Bedürfnisse und Barrieren beim gemeinsamen Leben und Lernen ins Gespräch zu kommen.

Der Bogen ist mit Blick auf mögliche Barrieren für Autist:innen entstanden, da Barrieren für diese Personengruppe teilweise unsichtbar sind und bislang häufig erst nach Eskalationen mühsam erschlossen werden mussten. Bei der Betrachtung der im Fragebogen genannten Beispiele wird deutlich, dass diese Situationen nicht nur für autistische Schüler:innen problematisch sein können. Das zeigen auch die Forschungsergebnisse.

Der schAUT-Barrierenfragebogen ist daher kein autismusspezifisches Instrument, sondern ein Hilfsmittel zu Inklusion und barrieresensibler Gestaltung des schulischen Umfelds und soll im Klassenverband eingesetzt werden. Das Vorliegen etwaiger klinischer Diagnosen oder festgestellter Förderschwerpunkte ist für die Identifikation möglicher Lern- und Teilhabebarrieren nicht relevant. Im Rahmen der Forschung und Fragebogenentwicklung des schAUT-Projekts wurde, für den Vergleich unterschiedlich vulnerabler Personengruppen, deshalb die Selbstauskunft bzw. anonymisierte Elternauskunft darüber zugrunde gelegt, ob die Teilnehmer:innen „autistisch“ oder „vielleicht autistisch“ sind.

Wichtig ist deshalb: Für die Anwendung des schAUT-Barrierenfragebogens sind klinische oder sonderpädagogische Diagnosen nicht von Belang. Die Ergebnisse des Fragebogens liefern diesbezüglich ebenfalls keinerlei Informationen.

Wir empfehlen, nach Möglichkeit nicht nur mit Klassen sondern mit ganzen Jahrgängen zu arbeiten, da die Schüler:innen einer Klassenstufe einen großen Teil der allgemeinen Lernumgebung teilen. Ähneln sich dabei zum Beispiel die Bereiche, die von vielen Schüler:innen als Hindernis identifiziert wurden, führt das ggf. zu anderen Priorisierungen bei der Entwicklung eines barrieresensiblen Umfelds.

Die Teilnahme an der Befragung sollte grundsätzlich freiwillig sein. Bitte sprechen Sie das Vorhaben auch in Ihrem Kollegium und mit der Schulleitung ab, um das Vorhaben barrieresensible Schulentwicklung bekannt zu machen und um ggf. datenschutzrechtliche Fragen abzustimmen. Wir empfehlen, die Eltern der beteiligten Klassen über das Schulentwicklungsvorhaben und die Befragung zu informieren und ggf. das Einverständnis der Eltern einzuholen. Eine Vorlage für einen möglichen Elternbrief finden Sie im Anhang dieses Manuals.

Befragung der Schüler:innen mit dem schAUT-Barrierenfragebogen

Die Befragung mit dem schAUT-Barrierenfragebogen sollte mindestens klassenweise bzw. nach Möglichkeit in noch übergreifenderen Gruppen (Klassenstufe, Schule) simultan stattfinden. Die Idee hierbei ist, ein umfassendes Bild über Bedürfnisse der Schüler:innenschaft zu bekommen, die große Teile ihres schulischen Alltags teilt. Die Auswertung erfolgt getrennt nach Klassen, so dass sich die Ergebnisse dann vergleichen lassen.

Umfeld für die Befragung

Mit dem schAUT-Barrierenfragebogen sollen die subjektiven Einschätzungen der Schüler:innen für Situationen erfragt werden. Es handelt sich demnach hierbei um ***keine standardisierte Testsituation***, sondern Sie möchten möglichst gute ***subjektive Urteile*** von den Schüler:innen erhalten. Der Kontext der Befragung sollte möglichst entspannt sein, damit die Schüler:innen nicht das Gefühl haben, dass sie sich in einer Unterrichts- oder Testsituation befinden. Wenn es sich anbietet, für die Befragung andere Räume als sonst zu nutzen oder nach draußen zu gehen, steht Ihnen das frei. Bei Verständnisschwierigkeiten der Kinder können Sie mit eigenen Worten die Beispiele erläutern oder auch manchmal alternative Beispiele anbieten, wenn die konkret beschriebene Situation so im Leben Ihrer Schüler:innen nicht vorkommt. Ein Beispiel hierfür wäre, die Eile nach dem Schwimmunterricht durch die Eile nach dem Sportunterricht oder in einer anderen Um- oder Ankleidesituation zu ersetzen. Oder die nicht regulierbare Essensauswahl beim Schulessen durch eine vergleichbare Situation auf der Klassenfahrt. ***Abweichungen vom Wortlaut sind also ausdrücklich erlaubt***.

Anonymisierung

Wichtig ist auch, dass die Befragung ***anonymisiert*** stattfindet, da es nicht das Ziel ist, einzelne Schüler:innen herauszustellen oder einzelne Bedürfnisse exklusiv zu erfüllen, sondern für die gesamte Klasse den Unterricht oder den Schulalltag barrieresensibler zu gestalten. Um das sicherzustellen, sollten die Fragebögen nach dem Ausfüllen nicht einzelnen Schüler:innen zuordenbar sein und dementsprechend vor dem Ausfüllen ggf. mit der Klassenbezeichnung, ***jedoch keinesfalls mit Namen beschriftet*** werden. Dies stellt den Schutz und die ***vertrauliche*** Behandlung der subjektiven Daten der Schüler:innen sicher.

Anleitung

Im ersten Schritt sollten Sie den für Ihre Schüler:innen passenden Fragebogen auswählen. Es gibt zwei Varianten, die inhaltlich grundsätzlich identisch sind. Sie unterscheiden sich jedoch in der grafischen Aufbereitung und im Umfang der Instruktionen.

Der Bogen, der mit ***Grundschule*** überschrieben ist, wird verwendet, wenn die Schüler:innen noch im Lese-Lern-Prozess sind. Hier müssen die Anweisungen und auch alle Beispiele ***vorgelesen*** werden. Um das Mitlesen zu unterstützen, wurden in den Beispielen einzelne Wörter durch Symbole ersetzt, wie das in manchen Kinderbüchern üblich ist. Im ***Anhang unter „Materialien“*** finden Sie die ***gesamte vorzulesende Instruktion***, die ***nicht im Grundschul-Fragebogen enthalten*** ist.

Der **Sekundarstufenbogen** ist für die höheren Klassen (ab Klasse 5) gedacht, Sie können diesen Bogen nutzen, wenn die Schüler:innen die Anweisungen und Beispiele auch selbst lesen können.

Bevor Sie mit dem Ausfüllen des Fragebogens beginnen, sollten Sie mit einigen Worten die Befragung einleiten. Für diese Einführung finden Sie im Anhang unter „Materialien“ Texte sowie die Bewertungsskala im Großformat. Sie kann ausgedruckt und vor Beginn an der Tafel

präsentiert werden. Unabhängig von der Klassenstufe ist es wichtig, die Bewertungsstufen einmal in der Gruppe durchzugehen, um die Bedeutung der Stufen zu erklären. Die Skala ist eingeteilt in 5 Stufen. Sie reichen von Stufe 1 („Das würde mich gar nicht stören") bis Stufe 5 („Das würde mich so sehr stören, dass ich überhaupt nichts mehr machen kann").

Sie sollten an einem Beispiel verdeutlichen, dass unterschiedlichste Urteile möglich sind und es keine falschen Antworten gibt.

Ein Beispiel:

„Das Lieblingseis im Eisladen ist ausverkauft. Wie sehr würde dich das stören?"

Typischerweise bekommt man die ganze Bandbreite an Urteilen und kann dementsprechend gut verdeutlichen, dass verschiedene Urteile möglich sind.

Wichtig ist hier auch, noch einmal klarzumachen, dass die Stufe 5 nur vergeben werden soll, wenn die Situation so schlimm ist, dass man wirklich überhaupt nichts mehr machen kann. Also schlimmstenfalls auch nicht sprechen, nicht denken und nicht verstehen.

Auf die Bedeutung der Skalenpunkte und auf die Abstufungen sollte im Befragungsverlauf wiederholt hingewiesen werden. Insbesondere im Grundschulbereich ist eine Unterstützung der Vorstellungskraft durch weitere Beschreibungen hilfreich, um besonders treffende Einschätzungen überhaupt möglich zu machen.

Generell gilt: Sie sind frei beim Erklären, müssen aber darauf achten, dass Sie ***das Urteil der Schüler:innen nicht beeinflussen***.

Auswertung des schAUT-Barrierenfragebogens

Eingabe der Daten

Im Anschluss an die Durchführung müssen die Daten aus den ausgefüllten Fragebögen in die Auswertungsdatei übertragen werden. Die automatisierte Auswertung zeigt Ihnen dann in Tabellen und Grafiken, welche Bereiche und speziellen Barrieren für Ihre Schüler:innen eine Rolle spielen. Sie benötigen dafür Excel aus dem Microsoft Office Paket in einer aktuellen Version (2010 oder neuer).

Bitte laden Sie dafür zunächst ***die Datei „schAUT_Daten.xlsx"*** von der Homepage der schAUT-Fortbildung (https://hu.berlin/schAUT, Modul 2) herunter, speichern Sie diese auf Ihrem Computer und benennen Sie sie so um, dass Sie die Zugehörigkeit zur jeweiligen Klasse wiedererkennen, z.B. in „schAUT_7a_Mai24.xlsx".

Wenn Sie die Datei öffnen, finden Sie auf dem ersten Tabellenblatt das „Dateneingabeblatt" (vgl. Abb. 1). Dort sehen Sie alle 50 Einzelitems des Fragebogens in der oberen Zeile aufgelistet. Um Ihnen bei der Eingabe die Orientierung zu erleichtern, sind in den Spalten auch noch mal die Bilder zu sehen, die das jeweilige Beispiel im Fragebogen illustrieren. In der ersten Spalte stehen Teilnehmer-Nummern, die Sie den ausgefüllten Fragebögen geben, so wie sie vom Stapel kommen. Die Reihenfolge ist dabei egal, da ja keine individuelle Zuordnung möglich sein soll. Die Ergebnisse der Fragebögen werden nun in die leeren Zellen in der Mitte übertragen. Bitte nutzen Sie für jeden Fragebogen eine eigene Zeile.

Wichtig:
Wenn ein Schüler oder eine Schülerin bei einer Frage kein Kreuz, mehrere Kreuze oder ein Kreuz zwischen zwei Zahlen gemacht hat, dann lassen Sie dieses Tabellenfeld bitte ***leer***.

Tragen Sie bitte auch ***keine 0 oder andere Zeichen/Buchstaben*** ein. Die automatische Auswertung der Tabelle funktioniert sonst nicht richtig und die Ergebnisse werden verfälscht.

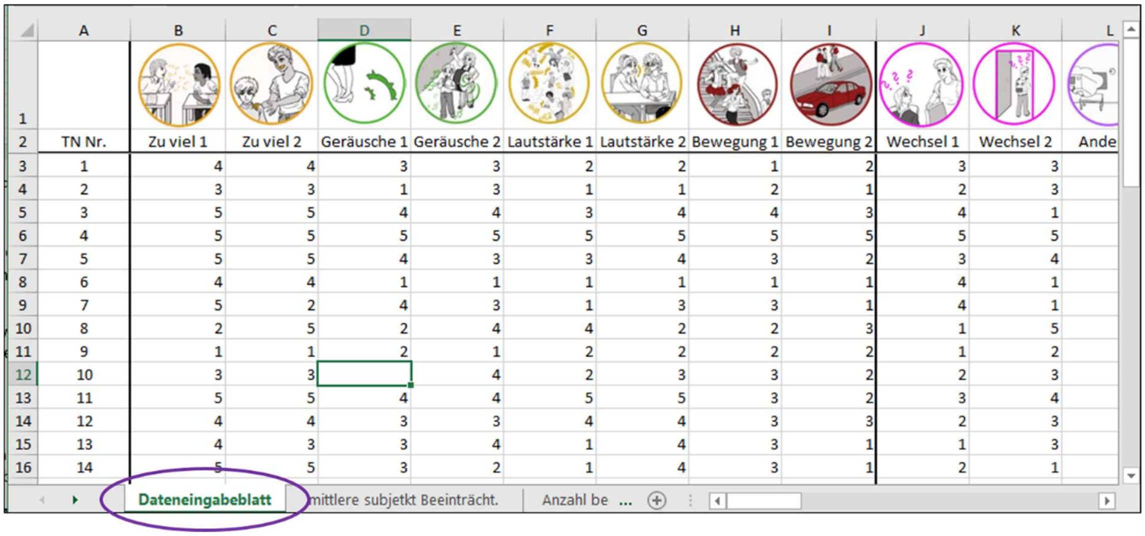

	A	B	C	D	E	F	G	H	I	J	K	L
1												
2	TN Nr.	Zu viel 1	Zu viel 2	Geräusche 1	Geräusche 2	Lautstärke 1	Lautstärke 2	Bewegung 1	Bewegung 2	Wechsel 1	Wechsel 2	Ande
3	1	4	4	3	3	2	2	1	2	3	3	
4	2	3	3	1	3	1	1	2	1	2	3	
5	3	5	5	4	4	3	4	4	3	4	1	
6	4	5	5	5	5	5	5	5	5	5	5	
7	5	5	5	4	3	3	4	3	2	3	4	
8	6	4	4	1	1	1	1	1	1	4	1	
9	7	5	2	4	3	1	3	3	1	4	1	
10	8	2	5	2	4	4	2	2	3	1	5	
11	9	1	1	2	1	2	2	2	2	1	2	
12	10	3	3		4	2	3	3	2	2	3	
13	11	5	5	4	4	5	5	3	2	3	4	
14	12	4	4	3	3	4	4	3	3	2	3	
15	13	4	3	3	4	1	4	3	1	1	3	
16	14	5	5	3	2	1	4	3	1	2	1	

***Abb. 1:** Beispiel für Daten im Dateneingabeblatt, mit einer fehlenden Antwort bei Teilnehmer:in 10*

Die Daten werden statistisch ausgewertet. Deshalb sollten nicht zu wenige Schüler:innen sondern eine hinreichend große Gruppe aus der Klasse teilgenommen haben, um ein möglichst repräsentatives Bild zu bekommen und um die nötige Anonymität der Daten zu gewährleisten.

Die beiden Beispiele auf einem Fragebogenblatt gehören jeweils zu einer Barriere. Im Rahmen der Auswertung werden die im Fragebogen untersuchten 25 potenziellen Barrieren wiederum acht Bereichen zugeordnet.

Informationen aus den Auswertungen

Wenn das Dateneingabeblatt ausreichend Daten enthält, werden auf den nachfolgenden Tabellenblättern der Excel-Datei automatisch folgende Angaben bereitgestellt:

- Die Anzahl an Schüler:innen in der Klasse, die insgesamt über alle 50 Situationen hinweg erhöhte Werte (größer als 3,5) angegeben haben (vgl. **Abb 2**).
- Den Mittelwert der Einschätzungen in der Klasse für die acht Barrierenbereiche (vgl. **Abb. 2**).
- Die Anzahl an Schüler:innen in der Klasse, die Situationen der acht Bereiche als stark beeinträchtigend (3 bis 5) oder kaum beeinträchtigend (1 oder 2) empfinden (vgl **Abb 3**).
- Für jeden der acht Bereiche, getrennt für die zugeordneten Barrieren: die Anzahl an Schüler:innen, welche die jeweilige Barriere als stark beeinträchtigend (3 bis 5) oder kaum beeinträchtigend (1 oder 2) empfinden (vgl. **Abb. 4**).

Mit Hilfe dieser Auswertung können Bereiche und Barrieren identifiziert werden, die innerhalb der jeweiligen Klasse relevant sind. Ein Vergleich der aktuellen Gestaltung des Schulalltags und der Lernumgebung mit den Untersuchungsergebnissen macht es möglich abzuschätzen, inwiefern die Bedürfnisse der Schüler:innen bereits berücksichtigt werden und an welchen Stellen noch Anpassungen notwendig sind. Umgekehrt können die Fragebogenergebnisse ggf. Erklärungsmöglichkeiten für schwierige Situationen in der Vergangenheit liefern, die durch Barrieren entstanden sein könnten. Die erkannte Anzahl an stark gefährdeten Schüler:innen bietet außerdem eine Information darüber, wie dringlich die Berücksichtigung der bestehenden Lernbarrieren in dieser Klasse ist.

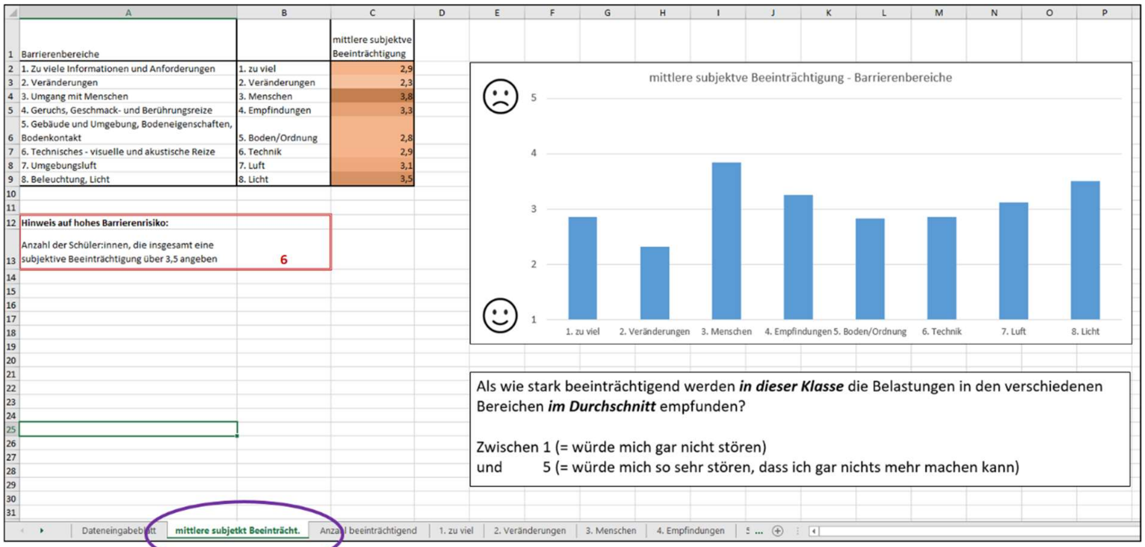

***Abb. 2**: Anzahl an Schüler:innen in der Klasse, die insgesamt über alle 50 Situationen hinweg erhöhte Werte (größer als 3,5) angegeben haben und Mittelwerte der Einschätzungen in der Klasse für die acht Barrieren-bereiche - Beispiel*

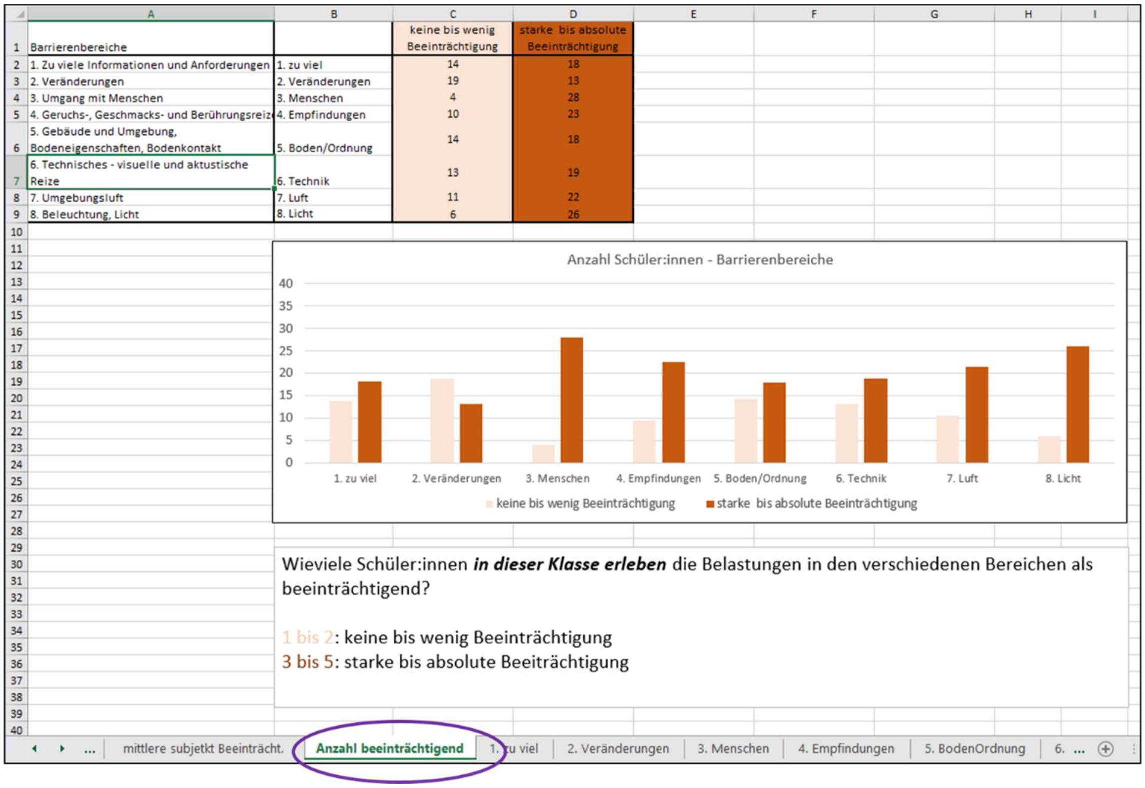

***Abb. 3:** Anzahl an Schüler:innen in der Klasse, die Situationen der acht Bereiche als stark beeinträchtigend (3 bis 5) oder kaum beeinträchtigend (1 oder 2) empfinden – Beispiel*

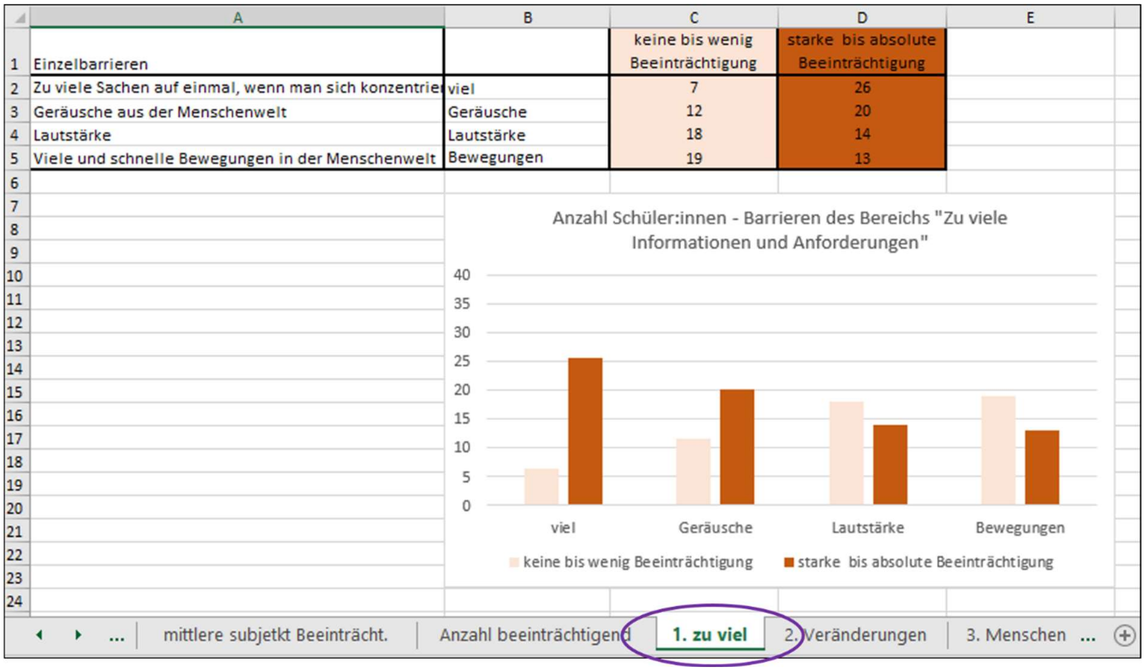

	A	B	C	D
1	Einzelbarrieren		keine bis wenig Beeinträchtigung	starke bis absolute Beeinträchtigung
2	Zu viele Sachen auf einmal, wenn man sich konzentrie	viel	7	26
3	Geräusche aus der Menschenwelt	Geräusche	12	20
4	Lautstärke	Lautstärke	18	14
5	Viele und schnelle Bewegungen in der Menschenwelt	Bewegungen	19	13

***Abb. 4:** Anzahl an Schüler:innen, welche die jeweilige Barrieren des Bereichs „Zu viele Informationen und Anforderungen" als stark beeinträchtigend (3 bis 5) oder kaum beeinträchtigend (1 oder 2) empfinden - Beispiel*

Nutzung der Ergebnisse für die Arbeit mit der Handreichung

Um den Abbau von Barrieren zu gestalten, wird es in den meisten Fällen notwendig sein, Prioritäten zu setzen, da nicht alle Barrieren auf einmal bearbeitet werden können.

Generell gilt: Wenn es Schüler:innen mit auffällig hohen Gesamtwerten gibt, sollte dringend auf mögliche Barrieren reagiert werden (je mehr, desto dringender)!

Eine Betrachtung der Mittelwerte in den acht Barrierenbereichen (Abb. 2) gibt Hinweise auf die Bereiche, in denen für die untersuchten Schüler:innen mit großer Wahrscheinlichkeit Barrieren entstehen. Jeder Bereich umfasst mehrere Barrieren, so dass für eine Priorisierung geprüft werden sollte, bei welchen Barrieren des Bereichs die meisten Schüler:innen erhöhte Werte angeben (vgl. Abb. 4).

Wählen Sie zur Besprechung im Team zwei oder höchstens drei Barrieren auf einmal aus. Priorisieren Sie die Bereiche, die in mehreren Klassen eine hohe Relevanz haben. Nur so ist es möglich, Maßnahmen zu erarbeiten, ihre Umsetzung gut zu begleiten und mittelfristig ihre Wirksamkeit einzuschätzen. Beginnen Sie die Diskussion zu einer Barriere immer mit einer Einschätzung, wie stark und an welchen Stellen die ausgewählte Barriere tatsächlich im Schulalltag zum Tragen kommt. Als Ausgangspunkt für die anschließende gemeinsame Erarbeitung möglicher Maßnahmen stehen Ihnen die Vorschläge in Kapitel 6 der Handreichung bzw. die Zusammenstellung der Vorschläge auf der Homepage zur Verfügung (https://hu.berlin/schAUT, Modul 3).

Zur Einschätzung, wie gut barrieresensible Gestaltung an Ihrer Schule gelingt und sich entwickelt, ist das Selbstevaluationsraster schAUT-S (schAUT Schulentwicklungscheck, https://hu.berlin/schAUT, Modul 4) geeignet. Da es in vier Niveaustufen unterscheidet, lassen sich hier sowohl Fortschritte als auch weiterer Handlungsbedarf gut sichtbar machen. Nähere Informationen dazu finden Sie in der Handreichung (Kapitel 8).

Der schAUT-Barrierenfragebogen ist weniger gut geeignet, um den Erfolg einer barrieresensiblen Gestaltung abzubilden, da er fragt, wie stark man persönlich auf eine Situation reagieren würde, egal ob sie vorhanden ist oder nicht. Eine Wiederholung der Befragung ist dann sinnvoll, ***wenn der Eindruck besteht, dass sich die Bedarfe der Schüler:innen durch ihre Entwicklung verändert haben***. Die Erhebungen im Rahmen des schAUT-Projekts haben ergeben, dass die möglichen Beeinträchtigungen durch Lernbarrieren in den ersten Schuljahren für die meisten Schüler:innen höher sind. In der Sekundarstufe ist das Niveau besonders für autistische Schüler:innen hoch. Dies deutet auf eine Veränderung im Laufe der Zeit hin. Ein wiederholter Einsatz in größerem Zeitabstand (ein Jahr oder mehr) ist daher empfehlenswert.

Entwicklung des Instruments und Güte des Barrierenfragebogens

Der schAUT-Barrierenfragebogen wurde in mehreren Stufen nach wissenschaftlichen Kriterien entwickelt. Dabei sind Vorarbeiten des White-Unicorn e.V., die Ergebnisse mehrerer Online-Vorstudien unter Einbeziehung der autistischen Community sowie die Daten aus 19 am schAUT-Projekt teilnehmenden Schulen in Berlin, Hessen und Nordrhein-Westfalen eingeflossen.

Welche Barrieren für Autist:innen gibt es?

Wenn für Autist:innen problematische Umstände erfasst werden sollen, ist es wichtig, vor allem die Erfahrungen autistischer Personen einzubeziehen, um keine potenziellen Barrieren zu übersehen. White-Unicorn e.V. hat 2018 eine Sammlung von Kontexten entwickelt, die für Autist:innen zu sensorischen oder sozialen Barrieren werden können. Grundlage dafür war die Auswertung einer Umfrage der Enthinderungsselbsthilfe (2008). Studienergebnisse zeigen, dass sich diese Barrierenliste eignet, die Barrierenlast mittels eines Fragebogens zu erfassen (White Unicorn e.V., 2018). In dieser Untersuchung war auf einer Skala anzugeben, wie stark man durch die beschriebene Barriere beeinträchtigt wäre. Die Daten bildeten für alle Barrieren zuverlässig Unterschiede zwischen Autist:innen und Nicht-Autist:innen ab. Diese Sammlung von Barrieren bildet die Grundlage des Schülerfragebogens des schAUT-Projekts.

Von den Barrieren zu Fragebogenitems

Die Beschreibungen der Barrieren wurden zunächst an den schulischen Kontext angepasst. In einer ersten Onlinestudie im Rahmen des schAUT-Projekts wurden die Teilnehmer:innen gebeten, Beispiele dafür zu nennen, wann die einzelnen Barrieren im schulischen Alltag auftreten können. 770 Personen formulierten offene Antworten zu den 27 möglichen Barrieren. Durch eine qualitative Inhaltsanalyse (Mayring, 2008) wurden für jede Barriere die vier am häufigsten beschriebenen Situationen ermittelt (Kleres et al., in prep.). Für zwei der Barrieren überschnitten sich diese Beispiele vollständig mit anderen, so dass diese Barrieren bei der Weiterentwicklung wegfielen. Tab. 1 enthält in der linken Spalte eine Liste der 25 Barrieren, eine ausführliche Beschreibung findet sich in Kapitel 6 der Handreichung (https://hu.berlin/schAUT, Modul 3).
Für 25 Barrieren wurden nun die vier ermittelten Beispielsituationen in kurzen Sätzen beschrieben und illustriert. In einer nächsten Onlinestudie waren diese 100 Beispiele (Fragebogenitems) auf einer fünftstufigen bipolaren Skala von „das fände ich prima“ bis „das stört mich so sehr, dass ich gar nichts mehr machen kann“ zu bewerten. Die Daten von 366 Kindern und Jugendlichen (max. 20 Jahre, davon laut Selbstauskunft in der Umfrage 191 autistisch und 97 vielleicht autistisch) wurden für die Weiterentwicklung des Barrierenfragebogens ausgewertet (Schwager et al., in prep.).

Barrieren	Barrierenbereich
• Zu viele Sachen auf einmal, wenn man sich konzentriert<br>• Geräusche aus der Menschenwelt<br>• Lautstärke<br>• Viele und schnelle Bewegungen in der Menschenwelt	1. Zu viele Informationen und Anforderungen
• Wenn man ständig die Umgebung und Räume wechseln muss<br>• Wenn bekannte Orte und Sachen plötzlich anders aussehen<br>• Viele neue unbekannte Formen und Sachen<br>• Unfertiges<br>• Wenn bestimmte Muster ein Problem sind<br>• Farbige Texte und Markierungen	2. Veränderungen
• Mitmenschen als Bedrohung<br>• Hast und Eile	3. Umgang mit Menschen
• Gerüche, die stören<br>• Ungewollter Geschmack<br>• Dinge, die sich schrecklich anfühlen<br>• Ungewollt zu nahe kommen	4. Geruch, Geschmack, Berührung
• Erschütterungen: Wenn der Boden zittert und die Wände wackeln<br>• Ungerader und ungleichmäßiger Boden<br>• Ordnung, weil in der Menschenwelt zu viele Hindernisse sind	5. Gebäude und Umgebung, Boden und Bodenkontakt
• Töne, die andere nicht mehr hören können<br>• Kunstlicht	6. Technisches (visuelle und akustische Reize)
• Luftbewegungen<br>• Temperaturunterschiede	7. Umgebungsluft
• Zu helles Licht<br>• Spiegelbilder und Reflexionen	8. Beleuchtung / Licht

***Tab. 1:** Liste der 25 Barrieren und 8 Barrierenbereiche*

Von 100 Fragebogenitems zu 50 Fragebogenitems und acht Barrierenbereichen

Die statistische Analyse der Zusammenhänge zwischen den Antworten (Hauptkomponentenanalyse) ergab, dass sich die Barrieren anhand der Items zu acht Bereichen (Komponenten) gruppieren lassen. Die Antworten auf die Items ein und desselben Bereichs fallen dabei ähnlicher aus als Antworten auf Items in anderen Bereichen. Tab. 1 enthält in der rechten Spalte die acht Barrierenbereiche zu denen die Barrieren gruppiert wurden.

Der Barrierenfragebogen sollte für eine handhabbare Anwendung in der Schule um die Hälfte, von 100 auf 50 Items, gekürzt werden. Die Auswahl der Items erfolgte nach mehreren statistischen und inhaltlichen Kriterien: (1) Die Items sollten möglichst eindeutig mit einem der acht Bereiche zusammenhängen[1], (2) sie sollen sich gut dafür eignen, das Ausmaß der Beeinträchtigung durch die Situation zu erfassen, d.h. gut zwischen Personen mit unterschiedlichen Ausprägungen differenzieren[2], (3) für jede der 25 Barrieren sollten zwei Items im Fragebogen verbleiben. Die statistische Analyse ergab außerdem, dass die beschriebenen Situationen nur sehr vereinzelt positiv („das finde ich prima“ oder „das finde ich ganz ok“) beantwortet wurden. Eine Skala, die lediglich in Stufen das Ausmaß der Beeinträchtigung (und nicht die positive Bewertung) erfasst, ist demnach deutlich geeigneter für den Fragebogen. Eine weitere Onlinestudie wurde mit dem auf 50 Items verkürzten Fragebogen und einer fünfstufigen unipolaren Skala von 1 „stört mich gar nicht“ bis 5 „stört so sehr, dass ich überhaupt nichts mehr machen kann“ durchgeführt. Die Daten von 614 Kindern und Jugendlichen wurden ausgewertet (bis max. 20 Jahre, davon 254 autistisch und 160 vielleicht autistisch). Bei der statistischen Überprüfung zeigten alle Items auch mit der neuen Skala eine gute Differenzierungsfähigkeit. Außerdem konnten die acht Barrierenbereiche durch eine konfirmatorische Faktorenanalyse auch mit dem verkürzten Itemsatz bestätigt werden[3].

[1] Items mit Querladungen (ähnlich hoher Zusammenhang mit mehreren Komponenten) und mit Ladungen <.3 wurden ausgeschlossen.

[2] Die Trennschärfe (Differenzierungsfähigkeit) bezogen auf den Summenwert des zugehörigen Barrierenbereichs sollte mindesten 0.4 sein.

[3] Gütekrierien für die Modellanpassung: TLI =.93; CFI=.94; RMSEA= .05 (p > .4)

Der aktuelle Schülerfragebogen enthält die erprobten 50 Items. Einzelne Items wurden in der letzten Version geringfügig sprachlich angepasst, um sie inhaltlich eindeutiger zu machen. In der Reihenfolge sind die Items nun nach den acht Barrierenbereichen geordnet, wobei sich die beiden Items, die zu einer Barriere gehören, immer auf derselben Seite befinden (bei Hochformat untereinander, bei Querformat nebeneinander).

Wissenschaftliche Gütekriterien für die finale Fragebogenversion

Der schAUT-Barrierenfragebogen ist ein psychometrisches Instrument – das heißt, man versucht, mit seiner Hilfe die Ausprägung der subjektiven Beeinträchtigung durch bestimmte Barrieren zu messen. Unterschiedlich starke Beeinträchtigung bildet sich in unterschiedlich hohen Zahlenwerten ab. Die Güte eines solchen Messinstruments lässt sich anhand verschiedener Kriterien überprüfen und beschreiben. Als Hauptgütekriterien für psychometrische Verfahren gelten die Objektivität, die Reliabilität und die Validität.

Die Objektivität bezeichnet die Unabhängigkeit des Messergebnisses von der durchführenden bzw. auswertenden Person – dies ist im Falle des schAUT-Barrierenfragebogens dann gegeben, wenn gewährleistet werden kann, dass die Teilnehmer:innen die Items verstehen und in ihrem subjektiven Urteil nicht beeinflusst werden. Die Anleitungen zur Durchführung und Auswertung der Befragung sollen dies unterstützen.

Die Reliabilität beschreibt die Messgenauigkeit des Instruments – das heißt Items, die die gleiche Art der Beeinträchtigung (dieselbe Barriere oder denselben Barrierenbereich) erfassen, sollten ähnliche Werte ergeben. Die statistischen Indices für den schAUT-Barrierenfragebogen sprechen diesbezüglich für eine sehr hohe Messgenauigkeit[4].

Validität ist dann gegeben, wenn das Instrument inhaltlich das erfasst, was es erfassen soll. Da sowohl die Definition der Barrieren als auch die Erarbeitung des Fragebogens unter Einbezug der Expertise der autistischen Community und in Kooperation mit autistischen Kolleg:innen erfolgte, ist von einer guten Inhaltsvalidität auszugehen – der schAUT Barrierenfragebogen beschreibt Situationen, die insbesondere für Autist:innen zur Barriere werden können. Dies wird zusätzlich von den Ergebnissen aller Studien des schAUT-Projekts unterstützt, die zeigen, dass vielleicht autistische und autistische Personen sich stärker durch die Barrieren beeinträchtigt fühlen als nicht autistische Personen. Abb. 5 zeigt dies anhand der Daten aus der 2. Erhebung an den Projektschulen für die acht Bereiche – die mittlere subjektive Beeinträchtigung ist in allen Bereichen für autistische Schüler:innen am höchsten.

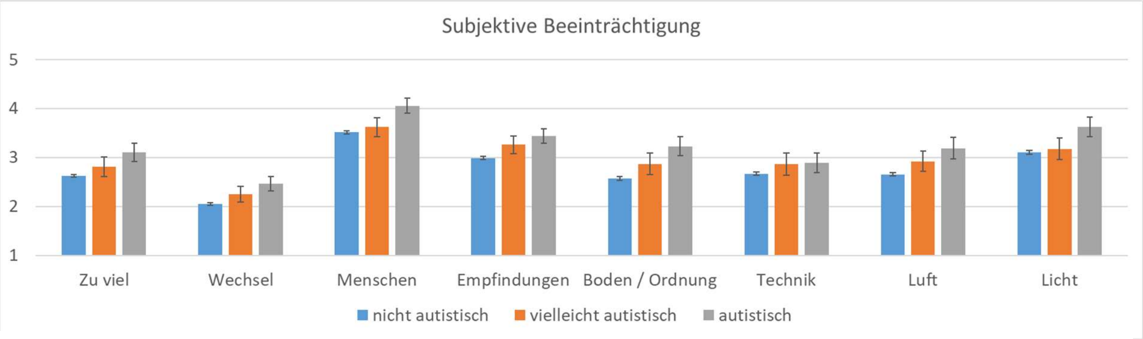

Abb. 5: *Mittlere subjektive Beeinträchtigung für die acht Barrierenbereiche und die Schüler:innengruppen bei der ersten schAUT-Befragung in den Schulen; Haupteffekt Gruppe F(2,813)=5.4; p=.005; d=.23*

[4] Testhalbierung: rtt = .93; Interne Konsistenz: Bereich 1 Zu viele Informationen und Anforderungen α = .83; Bereich 2 Veränderungen α = .79; Bereich 3 Umgang mit Menschen α =.76; Bereich 4 Geruch, Geschmack, Berührung α = .79; Bereich 5 Gebäude und Umgebung, Boden und Bodenkontakt α = .83; Bereich 6 Technisches α = .75; Bereich 7 Umgebungsluft α = .76; Bereich 8 Beleuchtung / Licht α = .81

Quellen

Enthinderungsselbsthilfe (2008). *Grundzüge der Kollision autistischer Eigenschaften mit nichtautistisch geprägter Umgebung*. Enthinderungsselbsthilfe. https://autisten.enthinderung.de/kollision/

Kleres, J., Benecke, M., Fuhrmann, S., Gerhards, L., Knigge, M, Moser, V. & Schwager, S. (in prep.). *Barriers for autistic school students - autists' perspectives*

Mayring, P. (2008). *Qualitative Inhaltsanalyse: Grundlagen und Techniken*. Weinheim: Beltz

Schwager, S. Benecke, M., Fuhrmann, S., Gerhards, L., Kleres, J., Knigge, M & Moser, V. (in prep.). *Identifying individual barriers of autistic children at school – development of a questionnaire as part of a tool to help inclusion practice in regular schools*

White Unicorn e.V. (2018). *Umfrage zu Barrieren für Autisten*. https://www.white-unicorn.org/autismus/barrieren

Materialien

Durchführungsanweisungen Grundschule

Materialien:

- Barrierenfragebogen-Heft
- ausgedruckte oder digitale Barrierenskala (Smileys und Kreise mit den Zahlen 1 bis 5 nebeneinander)

Vorbereitung:

Jedes Kind hat das Barrierenfragebogen-Heft sowie einen Stift vor sich liegen.

Allgemeine Informationen zur Durchführung:

Die im Folgenden kursiv geschriebenen Textpassagen sind Anweisungen für die Schüler:innen. Die Textpassagen in eckigen Klammern sind Handlungsempfehlungen für die durchführende Lehrkraft. Die Anweisungen sollten vollständig wiedergegeben werden, das muss jedoch nicht zwingend wortwörtlich sein. Wichtig ist, dass die Kinder den Inhalt verstehen. Dazu kann es hilfreich sein diesen zusätzlich zu paraphrasieren oder weitere Beispiele zu nennen.

Das Ausfüllen der Fragebögen wird ca. 45-60 Minuten in Anspruch nehmen.

Die Kinder sollen die Fragen allein und intuitiv beantworten. Wenn ein Kind Schwierigkeiten hat, sollten Sie darauf möglichst zügig reagieren und Hilfestellungen anbieten. Dabei sollten Sie darauf achten, dass Sie nicht Ihre eigene Einschätzung präsentieren, um eine Beeinflussung zu vermeiden.

Wenn ein Kind nicht weiterweiß, können Sie beispielsweise wie folgt reagieren**:** *„Ich darf dir dazu nicht mehr verraten. Überlege nochmal, wie sehr dich das stören würde und kreuze dann die Zahl an, die am besten dazu passt. Also stört es dich zum Beispiel so sehr, wenn [aktuelles Beispiel einsetzen], dass du gar nichts mehr machen kannst? Oder gar nicht? Oder etwas dazwischen?“*

Vermeiden Sie unbedingt Aussagen wie *„Das ist doch gar nicht so schlimm, oder?“*
Die Hilfestellung dient lediglich dazu, die Selbstreflexion der Schüler:innen anzuregen und beim Verständnis der Skalen zu unterstützen.

Falls Sie merken, dass die Kinder sich nicht mehr konzentrieren können, legen Sie eine kleine Pause ein, lassen Sie die Kinder ihre Hände ausschütteln, o.Ä..

In dieser Pause sollten die Hefte verdeckt auf dem Tisch liegen bleiben und sich nicht gegenseitig gezeigt werden. Die Aufsichtsperson sollte während des gesamten Ausfüllens anwesend sein und für Fragen bereitstehen und die Kinder ggf. ermuntern und loben!

Wörtliche Anweisungen Grundschule

Wir möchten, dass in unserer Schule alle Kinder gut lernen können. Das geht besser, wenn man sich wohlfühlt und einen nicht so viele Dinge stören. Und wir als Erwachsene möchten etwas dafür tun, dass es von diesen Dingen weniger in unserer Schule gibt. Jeder von uns und von euch erlebt aber seinen Schultag anders, und es sind für jeden andere Dinge, die stören. Bevor wir loslegen und uns Sachen überlegen können, müssen wir deshalb herausfinden, was am meisten stört. Deshalb fragen wir euch, denn um Euern Schulalltag geht es ja.

Eure Antworten werden für die ganz Klasse zusammen ausgewertet, und zwar anonym – bitte schreibt also keine Namen auf die Hefte, das ist ganz wichtig! Wir wollen wissen, was stört, aber nicht, was wer geantwortet hat. Darauf könnt ihr euch verlassen.

[Bitte heften Sie jetzt die Blätter für die Barrierenskala (Smileys und Kreise mit den Zahlen 1 bis 5 nebeneinander) an die Tafel.]

Hier geht es um Sachen, die einen stören. Stellt euch vor, ihr geht in euern Eisladen, und es gibt euer Lieblingseis nicht. Damit ihr zeigen könnt, wie sehr euch etwas stört oder nicht stört, habe ich etwas vorbereitet. Hier seht ihr die Antwortmöglichkeiten, wie sie nachher auch im Heft sind. Ihr könnt zwischen 1 und 5 auswählen, um zu zeigen, wie sehr euch etwas stört. [Auf die Skala mit den beiden Smileys zeigen.]

Für manche wäre es vielleicht ganz schrecklich, wenn es das Lieblingseis nicht gibt, weil alle anderen Sorten scheußlich schmecken und man großen Hunger hat. [Auf die 5 zeigen]. ***Und andere Kinder wiederum würde das nicht sehr stören, weil sie auch viele andere Eissorten mögen.*** [auf die 2 zeigen].

Wichtig - Bei Sachen, die einen stören, gibt es kein richtig oder falsch. Manche Sachen stören den einen total. Gleichzeitig stört das eine andere Person überhaupt nicht. So ist das eben. Wen würde es denn gar nicht stören, wenn es das Lieblingseis nicht gibt [Auf die 1 zeigen]***? Und wen fast nicht?*** [Auf die 2 zeigen] ***Und wen würde es schon stören?*** [Auf die 3 zeigen] ***Und wer wäre richtig genervt?*** [Auf die 4 zeigen] ***Und für wen, wäre das so schlimm, dass er gar nichts mehr machen kann – nicht mehr denken oder sprechen oder zuhören?*** [Auf die 5 zeigen]***. Ihr seht, alle sehen das unterschiedlich! Und das ist genau das, was wir hier wissen wollen. Es gibt keine richtigen oder falschen Antworten. Es geht nur darum, wie es euch selbst geht.***

Auch in der Schule gibt es Sachen, die stören. Deshalb wird in dem Heft für viele verschiedene Dinge in der Schule gefragt, wie sehr sie euch stören.

Bitte schlagt jetzt das Heft auf und lasst uns mit einem Beispiel anfangen: Kim fragt „Wie sehr würde dich das stören? Ich mache während der Stillarbeit die ganze Zeit Lärm.“ [Wählen Sie zur Demonstration z.B. Stampfen, Husten, lautes Zuklappen eines Buches, Schlüssel klappern. Zeigen Sie die Bewertungen an der Tafel]

- ***Manche Kinder würde das gar nicht stören. Wenn es Dich nicht stören würde, dann kreuze 1 an.*** [Auf die 1 zeigen]
- ***Manche Kinder würde das mehr stören, aber sie könnten trotzdem lernen. Wenn es bei Dir so ist, dann kreuze 2 an.*** [Auf die 2 zeigen]
- ***Wenn es Dich doch beim Lernen stören würde, dann kreuze 3 an.*** [Auf die 3 zeigen]
- ***Wenn es Dich sehr stören würde und Du gar nicht mehr aufpassen könntest, dann kreuze die 4 an.*** [Auf die 4 zeigen]
- ***Wenn es Dich so sehr stören würde, dass Du gar nichts mehr machen könntest, also auch nicht mehr malen oder spielen, dann kreuze die 5 an.*** [Auf die 5 zeigen]

[Warten Sie, bis die Kinder ihr Kreuz auf der Übungsseite gesetzt haben. Ggf. ein Ritual vereinbaren (Stift hinlegen, hochhalten o.ä.), um zu sehen, dass alle fertig sind.]

Alles klar? [Ggf. Fragen beantworten] ***Dann geht es jetzt los. Ich lese euch immer ein Beispiel vor, und jeder macht für sich ein Kreuz, wie sehr das stören würde. Kreuzt einfach an, wie sehr es euch stören würde.***

Manche Sachen kommen vielleicht bei euch gar nicht vor. Dann kreuzt an, wie schlimm es wäre, wenn es doch mal so wäre. Wenn ihr etwas nicht versteht, könnt ihr immer fragen. Und nochmal: es gibt keine richtigen oder falschen Antworten, man muss also nicht lange nachdenken.

[Nach Gefühl sollten die Bedeutungen der Stufen der Skala während der Durchführung noch mehrmals wiederholt werden.]

Blättert jetzt bitte um.

Auf der nächsten Seite steht: Ich möchte im Unterricht zuhören, aber die anderen sind am Quatschen. Wie sehr würde Dich das stören?

Würde Dich das gar nicht stören? Oder ganz wenig? Oder etwas mehr? Sehr? Oder wäre es so schrecklich, dass Du überhaupt nichts mehr machen kannst? [die Skala zeigen]

Bitte kreuze jetzt beim ersten Bild an, wie sehr es Dich stören würde.

[wenn alle ihr Kreuz gesetzt haben, geht es weiter]

Beim zweiten Bild steht: Jemand stört mich mitten in einer Aufgabe und will etwas von mir. ***Wie sehr würde Dich das stören?***

Würde Dich das gar nicht stören? Oder ganz wenig? Oder etwas mehr? Sehr? Oder wäre es so schrecklich, dass Du überhaupt nichts mehr machen kannst? [die Skala zeigen]

Bitte kreuze jetzt beim zweiten Bild an, wie sehr es Dich stören würde.

[wenn alle ihr Kreuz gesetzt haben, geht es weiter]

Auf der nächsten Seite geht es weiter. Beim ersten Bild steht**….. [etc.]**

[Wiederholen Sie dies so lange, bis alle 25 Seiten vorgelesen und mit Kreuzen versehen sind.

Sie müssen die Beschreibung der Skalenpunkte nicht bei jedem Beispiel wiederholen, sollten aber immer mal wieder auf die fünf Abstufungen hinweisen. Sammeln Sie anschließend alle Hefte ein.

Sie dürfen die Beispiele bildhafter schildern oder alternative Situationen ähnlichen Inhalts finden (z.B. Eile beim Umziehen in einer anderen Situation als beim Schwimmunterricht), wenn die Situationen im Alltag der Kinder nicht vorkommen und die Abstraktion schwierig ist.

Vor dem Einsammeln ist es gut, die Kinder noch einmal durchblättern zu lassen, um zu schauen, ob sie bei jedem Bespiel ein Kreuz gesetzt haben.]

Durchführungsanweisungen Sekundarstufe

Materialien:

- Barrierenfragebogen-Heft
- ausgedruckte oder digitale Barrierenskala (Smileys und Kreise mit den Zahlen 1 bis 5 nebeneinander)

Vorbereitung:

Jede:r Schüler:in hat das Barrierenfragebogen-Heft sowie einen Stift vor sich liegen.

Allgemeine Informationen zur Durchführung:

Die im Folgenden kursiv geschriebenen Textpassagen sind Anweisungen für die Schüler:innen. Die Textpassagen in eckigen Klammern sind Handlungsempfehlungen für die durchführende Lehrkraft. Die Anweisungen sollten vollständig wiedergegeben werden, das muss nicht wortwörtlich sein.

Das Ausfüllen der Fragebögen wird ca. 30-45 Minuten in Anspruch nehmen.

Die Schüler:innen sollen die Fragen allein und intuitiv beantworten. Wenn es Schwierigkeiten gibt, sollten Sie möglichst zügig eine Hilfestellung anbieten. Dabei ist darauf zu achten, dass Sie nicht Ihre eigene Einschätzung präsentieren, um eine Beeinflussung zu vermeiden.

Wenn jemand nicht weiterweiß, können Sie beispielsweise wie folgt reagieren**:** *„Ich darf dir dazu nicht mehr verraten. Überlege nochmal, wie sehr dich das stören würde und kreuze dann die Zahl an, die am besten dazu passt. Also stört es dich zum Beispiel so sehr, wenn [aktuelles Beispiel einsetzen], dass du gar nichts mehr machen kannst? Oder gar nicht? Oder etwas dazwischen?“*

Vermeiden Sie unbedingt Aussagen wie *„Das ist doch gar nicht so schlimm, oder?“*
Die Hilfestellung dient lediglich dazu, die Selbstreflexion der Schüler:innen anzuregen und zum Skalenverständnis.

Falls Sie merken, dass die Kinder sich nicht mehr konzentrieren können, legen Sie eine kleine Pause ein. In der Pause sollten die Hefte verdeckt auf dem Tisch liegen bleiben und sich nicht gegenseitig gezeigt werden. Die Aufsichtsperson sollte während des gesamten Ausfüllens anwesend sein und für Fragen bereitstehen und ggf. ermuntern und loben!

Wörtliche Anweisungen Sekundarstufe

Wir möchten, dass in unserer Schule alle Schüler:innen gut lernen können. Das geht besser, wenn man sich wohlfühlt und einen nicht so viele Dinge stören. Und wir als Erwachsene möchten etwas dafür tun, dass es von diesen Dingen weniger in unserer Schule gibt. Jeder von uns und von euch erlebt aber seinen Schultag anders, und es sind für jeden andere Dinge, die stören. Bevor wir loslegen und uns Sachen überlegen können, müssen wir deshalb herausfinden, was am meisten stört. Deshalb fragen wir euch, denn um euern Schulalltag geht es ja.

Eure Antworten werden für die ganz Klasse zusammen ausgewertet, und zwar anonym – bitte schreibt also keine Namen auf die Hefte, das ist ganz wichtig! Wir wollen wissen, was stört, aber nicht, was wer geantwortet hat. Darauf könnt ihr euch verlassen.

[Bitte heften Sie jetzt die Blätter für die Barrierenskala (Smileys und Kreise mit den Zahlen 1 bis 5 nebeneinander) an die Tafel.]

Hier geht es um Sachen, die einen stören. Stellt euch vor, ihr geht in euern Eisladen, und es gibt euer Lieblingseis nicht. Damit ihr zeigen könnt, wie sehr euch etwas stört oder nicht stört, habe ich etwas vorbereitet. Hier seht ihr die Antwortmöglichkeiten, wie sie nachher auch im Heft sind. Ihr könnt zwischen 1 und 5 auswählen, um zu zeigen, wie sehr euch etwas stört. [Auf die Skala mit den beiden Smileys zeigen.]

Für manchen wäre es vielleicht ganz schrecklich, wenn es das Lieblingseis nicht gibt, weil alle anderen Sorten scheußlich schmecken und man großen Hunger hat. [Auf die 5 zeigen]. ***Und andere Kinder wiederum würde das nicht sehr stören, weil sie auch viele andere Eissorten mögen.*** [auf die 2 zeigen].

Wichtig - Bei Sachen, die einen stören, gibt es kein richtig oder falsch. Manche Sachen stören den einen total. Gleichzeitig stört das eine andere Person überhaupt nicht. So ist das eben. Wen würde es denn gar nicht stören, wenn es das Lieblingseis nicht gibt [Auf die 1 zeigen]***? Und wen fast nicht?*** [Auf die 2 zeigen] ***Und wen würde es schon stören?*** [Auf die 3 zeigen] ***Und wer wäre richtig genervt?*** [Auf die 4 zeigen] ***Und für wen, wäre das so schlimm, dass er gar nichts mehr machen kann – nicht mehr denken oder sprechen oder zuhören?*** [Auf die 5 zeigen]***. Ihr seht, alle sehen das unterschiedlich! Und das ist genau das, was wir hier wissen wollen. Es gibt keine richtigen oder falschen Antworten. Es geht nur darum, wie es euch selbst geht.***

Auch in der Schule gibt es Sachen, die stören. Deshalb wird in dem Heft für viele verschiedene Dinge in der Schule gefragt, wie sehr sie euch stören.

Wenn ihr das Heft aufmacht, seht ihr Kim. Kim erklärt euch wie es geht. Lest euch bitte Kims Beispiel durch und setzt euer Kreuz.

Gibt es Fragen? [Ggf. Fragen beantworten.]

Ihr findet jeweils ein Bild zu jeder Situation und einen Satz, der euch sagt, worum es geht. Bitte lest den Satz genau, überlegt kurz wie das für euch wäre, und kreuzt dann an, wie sehr euch die beschriebene Situation stören würde.

Alles klar? [Ggf. Fragen beantworten.]

Dann dürft ihr jetzt loslegen. Denkt daran, es gibt keine richtigen oder falschen Antworten. Ihr müsst euch also keine Sorgen machen. Kreuzt einfach jeder für sich allein an, wie es für euch wäre.

Macht für jede Situation nur ein Kreuz. Also entscheidet euch für eine Zahl.

Es gibt insgesamt 25 Seiten, die ihr jetzt so wie eben bitte in Stillarbeit bearbeitet.

[Vor dem Einsammeln ist es gut, die Schüler:innen noch einmal durchblättern zu lassen, um zu schauen, ob sie bei jedem Bespiel ein Kreuz gesetzt haben.]

Demonstrationsvorlage Skala

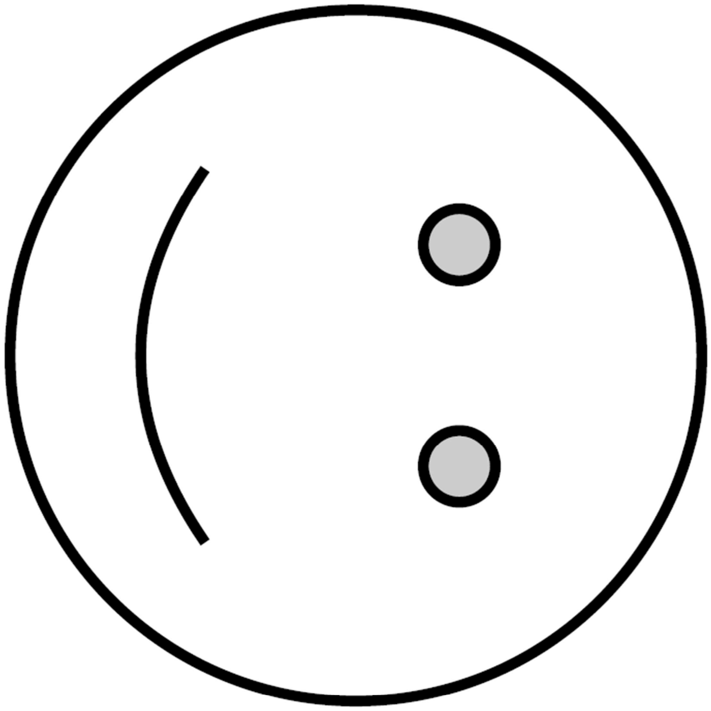

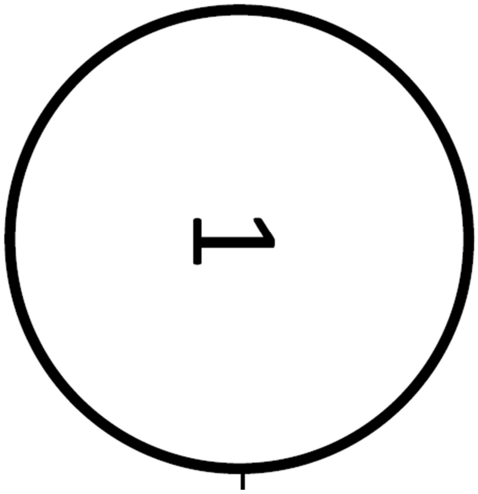

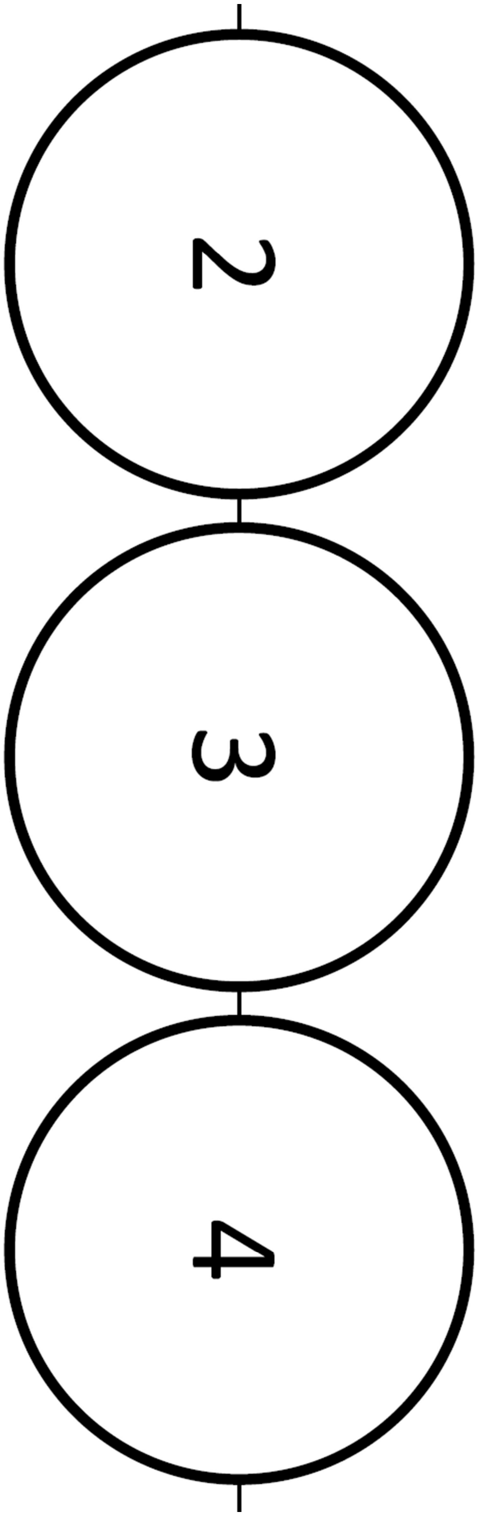
2
3
4

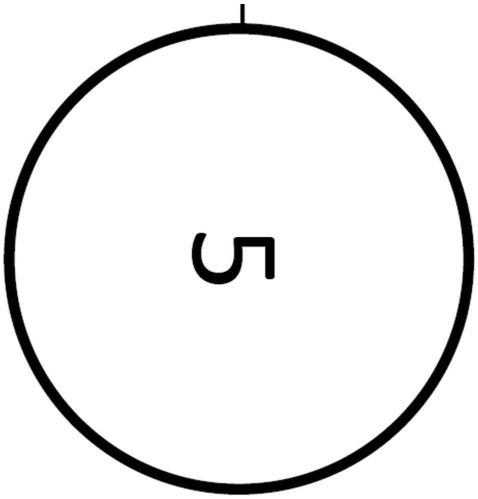
5

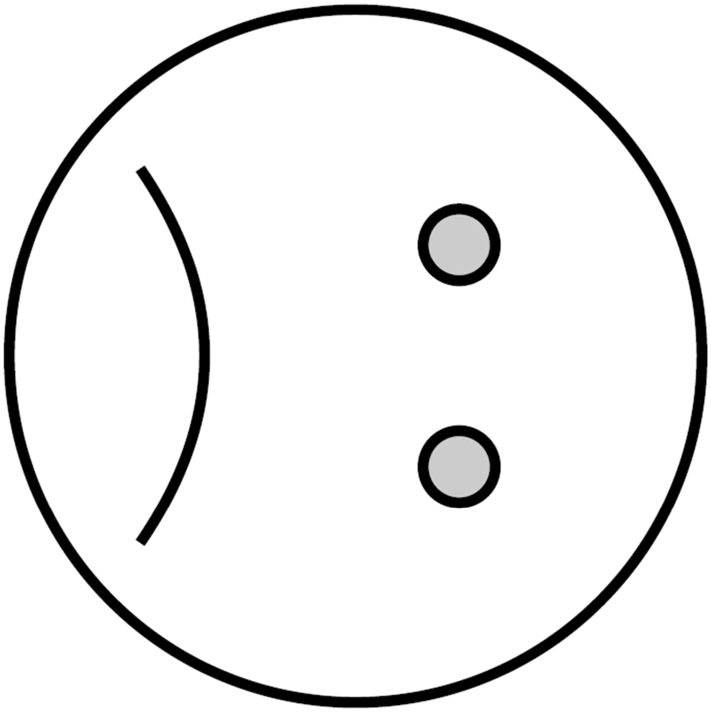

Vorlage Elternbrief

Liebe Eltern,

wir möchten Sie darüber informieren, dass wir in der Klasse Ihres Kindes eine **Umfrage mit einem Fragebogen** durchführen wollen. Ziel dieser Umfrage ist es, **Barrieren zu identifizieren, die möglicherweise den Schulalltag und das Lernen erschweren** und dadurch den Bildungserfolg beeinträchtigen können.

Der Fragebogen wurde im Rahmen eines Forschungsprojekts der Humboldt-Universität zu Berlin, der Goethe Universität Frankfurt und des White Unicorn e.V. entwickelt. Das Projekt wurde durch das Bundesministerium für Bildung und Forschung gefördert. Das Projekt ist inzwischen abgeschlossen und die Materialien, die in dem Forschungsprojekt entwickelt wurden, können in den Schulen angewendet werden. Wenn Sie gerne mehr über das Projekt erfahren wollen, können Sie sich auf der Projektwebsite umschauen: http://www.schaut-verbund.de/

Der Fragebogen ist dazu da, potenzielle Hindernisse in Schulen zu erkennen und Maßnahmen zur barrieresensiblen Schulgestaltung zu entwickeln, damit möglichst alle Kinder barrierearm am Unterricht teilhaben können. Dafür wird **in dem Fragbogen abgefragt, wie sehr bestimmte Situationen die Schüler:innen stören würden**, wie z.B. Lautstärke oder zu helles Licht.

Die Teilnahme an der Umfrage ist **für alle Kinder freiwillig**. Die Fragebögen werden **anonym** von den Lehrkräften der Schule ausgewertet und nicht an Dritte weitergegeben. Die Befragung dient einzig der barrieresensiblen Gestaltung innerhalb der Schule.

Wenn Sie damit einverstanden sind, dass Ihr Kind an der Umfrage teilnimmt, dann füllen Sie bitte die Einverständniserklärung aus und geben diese Ihrem Kind bis zum [Datum eintragen] wieder mit.

Mit freundlichen Grüßen,

[Name der Klassenleitung]

———————————————————————————————————————

Einverständniserklärung

Hiermit erkläre ich mich damit einverstanden, dass mein Kind ______________________________ an der Befragung zu möglichen Barrieren an der Schule teilnimmt.

__________________ ____

Datum Unterschrift

schAUT

Schule und Autismus - Diagnose von Barrieren für autistische Schüler:innen in inklusiven Schulen

White Unicorn
Verein zur Entwicklung eines autistenfreundlichen Umfeldes e.V.
Dr. Mark Benecke & Stephanie Fuhrmann
Hultschiner Damm 148 · 12623 Berlin
info@white-unicorn.org

Humboldt-Universität zu Berlin
Institut für Rehabilitationswissenschaften
Prof. Dr. Michel Knigge, Dr. Jochen Kleres, Jana Kunert & Dr. Sabine Schwager
Unter den Linden 6, 10099 Berlin

Goethe-Universität Frankfurt
FB Erziehungswissenschaften
Prof. Dr. Vera Moser & Lukas Gerhards
Theodor-W.-Adorno-Platz 6, 60323 Frankfurt am Main

Dieses Manual ist auch abrufbar unter https://hu.berlin/schAUT

Bundesministerium für Bildung und Forschung
Fördernummer: 01NV2104
